# C9ORF72 Deficiency Results in Neurodegeneration in the Zebrafish Retina

**DOI:** 10.1523/JNEUROSCI.2128-23.2024

**Published:** 2024-04-24

**Authors:** Natalia Jaroszynska, Andrea Salzinger, Themistoklis M. Tsarouchas, Catherina G. Becker, Thomas Becker, David A. Lyons, Ryan B. MacDonald, Marcus Keatinge

**Affiliations:** ^1^Institute of Ophthalmology, University College London, London EC1Y 0AD, United Kingdom; ^2^Centre for Clinical Brain Sciences, University of Edinburgh, Edinburgh EH16 4SB, United Kingdom; ^3^UK Dementia Research Institute at University of Edinburgh, University of Edinburgh, Edinburgh EH16 4SB, United Kingdom; ^4^Department of Psychiatry and Behavioural Sciences, Stanford University School of Medicine, Palo Alto, California 94305; ^5^Center for Regenerative Therapies Dresden (CRTD), Dresden 01307, Germany; ^6^Centre for Discovery Brain Sciences, University of Edinburgh, Edinburgh BioQuarter, Edinburgh EH16 4SB, United Kingdom

**Keywords:** ALS, C9ORF72, FTD, neurodegeneration, zebrafish

## Abstract

Hexanucleotide repeat expansions within the gene *C9ORF72* are the most common cause of the neurodegenerative diseases amyotrophic lateral sclerosis (ALS) and frontotemporal dementia (FTD). This disease-causing expansion leads to a reduction in C9ORF72 expression levels in patients, suggesting loss of C9ORF72 function could contribute to disease. To further understand the consequences of C9ORF72 deficiency in vivo, we generated a *c9orf72* mutant zebrafish line. Analysis of the adult female spinal cords revealed no appreciable neurodegenerative pathology such as loss of motor neurons or increased levels of neuroinflammation. However, detailed examination of adult female *c9orf72^−/−^* retinas showed prominent neurodegenerative features, including a decrease in retinal thickness, gliosis, and an overall reduction in neurons of all subtypes. Analysis of rod and cone cells within the photoreceptor layer showed a disturbance in their outer segment structure and rhodopsin mislocalization from rod outer segments to their cell bodies and synaptic terminals. Thus, C9ORF72 may play a previously unappreciated role in retinal homeostasis and suggests C9ORF72 deficiency can induce tissue specific neuronal loss.

## Significance Statement

Hexanucleotide expansions in the gene *C9ORF72* are the most common cause of the amyotrophic lateral sclerosis (ALS)/frontotemporal dementia (FTD) disease spectrum. The expansion reduces expression of C9ORF72 and so may play a role in neuronal loss. However, C9ORF72 loss of function has been comparatively understudied in vivo. Using the zebrafish as a model of C9ORF72 deficiency, we demonstrate that loss of C9ORF72 results in marked inflammation and neuronal loss in the aged adult zebrafish retina. Development of the retina is unaffected regardless of C9ORF72 status. This demonstrates that C9ORF72 loss of function can cause spontaneous neurodegeneration in vivo and highlights a novel role of C9ORF72 in retinal homeostasis.

## Introduction

Hexanucleotide GGGGCC (G_4_C_2_) repeat expansions within the first intron of *C9ORF72* are now recognized as the most common genetic cause of the neurodegenerative disease spectrum amyotrophic lateral sclerosis (ALS)/frontotemporal dementia (FTD; DeJesus-Hernandez et al., 2011; Renton et al., 2011). *C9ORF72* expansion mutations account for ∼40% of familial ALS cases (∼10% sporadic) and ∼25% of familial FTD cases (∼6% sporadic) depending on the population analyzed (Majounie et al., 2012). Several different mechanisms have been suggested to explain how these repeat expansions cause neuronal loss in the ALS/FTD disease spectrum which include both toxic gain of function (GoF) and loss of function (LoF). GoF mechanisms proposed to be pathogenic in disease include accumulation of toxic RNA foci and/or dipeptide repeats (DPRs; Balendra and Isaacs, 2018). The proposed LoF mechanism is the downregulation of C9ORF72 expression itself due to hypermethylation of its own promoter (Balendra and Isaacs, 2018; Braems et al., 2020). Although GoF mechanisms have been widely studied in vivo, LoF has been comparatively underexplored, with the function of *C9ORF72* still not completely understood (Balendra and Isaacs, 2018). In vitro studies suggest it to be part of the endosomal signaling network and to play a role in autophagy (Farg et al., 2014; Webster et al., 2016). In vivo, knock-out (KO) mouse models suggest a role for *C9ORF72* in the immune system with KO mice robustly exhibiting activation of macrophages and microglia in the absence of motor neuron (MN) loss; however, *C9ORF72* deficiency has been poorly explored in other in vivo animal models (Burberry et al., 2016; O’Rourke et al., 2016; Balendra and Isaacs, 2018). Therefore, it is unknown whether *C9ORF72*^−/−^ mutant mice are simply impervious to neuronal loss or whether the latter is a general feature of vertebrate *C9ORF72* deficiency in vivo. Genetic mouse models of other neurodegenerative conditions such as Parkinson’s disease have been shown to be resistant to neuronal loss compared with other vertebrate species such as rats and zebrafish (Kitada et al., 2009; Flinn et al., 2013; Dave et al., 2014). Furthermore how C9ORF72 deficiency impacts other parts of the CNS, such as the retina, has yet to be explored in vivo, despite ocular defects being a feature of ALS patients (Ringelstein et al., 2014; Mukherjee et al., 2017; Cerveró et al., 2021).

Complementary investigations using alternative C9ORF72-deficient vertebrate model species would build a more comprehensive understanding of C9ORF72 function in vivo but also to determine whether C9ORF72 deficiency can cause neuronal loss. The zebrafish is a promising complementary in vivo vertebrate model of neurodegeneration, demonstrating the key markers of pathology, including neuronal loss and gliosis, even during early larval stages, in models of various neurodegenerative conditions (Paquet et al., 2009; Flinn et al., 2013; Moreau et al., 2014; Keatinge et al., 2015, 2023; McGown et al., 2016; Lopez et al., 2017; Shaw et al., 2018; Larbalestier et al., 2022). Previous studies characterizing *c9orf72*-deficient larval zebrafish utilized a knockdown approach with Morpholinos (Ciura et al., 2013) and indicated that knockdown of *c9orf72* in zebrafish resulted in axon outgrowth deficits in MNs and a reduction in spontaneous movement (Ciura et al., 2013). However, the use of Morpholinos to study gene function in zebrafish neurons is difficult to interpret due to their potential off-target neurotoxic effects (Robu et al., 2007). Furthermore, adult *c9orf72* stable mutant zebrafish lines have yet to be described in detail, especially at late-stage adult ages when ALS/FTD is normally diagnosed in humans (Hruscha et al., 2013; Balendra and Isaacs, 2018).

Here we used CRISPR/Cas9 to generate a *c9orf72*-deficient zebrafish and characterized two separate components of the central nervous system, namely, the spinal cord and the retina. Similar to KO mice, *c9orf72*^−/−^ aged zebrafish do not show MN loss within the spinal cord. However, analysis of the retina unexpectedly revealed prominent disruption of overall structure with a large decrease of retinal thickness within the aged mutants, which has been reported in ALS patients (Moss et al., 2012; Fawzi et al., 2014). Most notably, *c9orf72^−/−^* exhibited a marked neuroinflammatory profile and significant reductions in key neuronal subtypes throughout the retina. Analysis of all retinal markers at larval stages revealed no differences between wild-type sibling control (WTS) and *c9orf72^−/−^*, suggesting the phenotypes observed here are due to progressive retinal degeneration in adults and not a developmental defect. Thus, the zebrafish represents a novel model to explore spontaneous neurodegeneration in a CNS tissue in C9ORF72 deficiency in vivo.

## Materials and Methods

### Zebrafish husbandry

All adult zebrafish were raised in standard conditions under project license PP5258250. The *c9orf72* mutant line was generated using CRISPR/Cas9 as previously described (Keatinge et al., 2021; Larbalestier et al., 2022) using standard Tracr RNA and a CrRNA targeting an *mwo1* restriction enzyme site within exon 2 of the zebrafish *c9orf72* (5′UAGAAGCUAAGCUCUGACUG), both purchased from Merck KGaA. This complex was coinjected with Cas9 protein (M0386M, NEB) into the yolk of one-cell stage WT zebrafish embryos. Once mutagenesis had been confirmed by restriction digest, F0 individuals were raised to adulthood and founders identified through crossing to WT. By using simple band shift on a standard agarose gel after PCR of the locus, a founder transmitting a large indel mutation to its F1 was identified. The *c9orf72* target locus was sequenced to confirm the mutation produced a frameshift and then propagated to establish a working colony of *c9orf72* heterozygous individuals. For 2 year old adult experiments, only females were analyzed due to a large sex skew in clutches.

### Genotyping and QPCR validation

The *c9orf72* mutant line was genotyped used primers F 5′ CTTCGTCTTGGCTGAAAAGG and R 5′ TTGTAACCCTAGAAGAAAAACACAAA and genotypes assigned from simple band shift, due to the size of the mutation, on a 2% agarose gel. Transcript levels were determined by QPCR using primers for *c9orf72* (F 5′ GCTTCTACCTGCCTCTGCAC and R 5′ATCAACCTCCTCTGGCACAC) and normalized to the housekeeping gene *ef1a* (F 5′ TGGTACTTCTCAGGCTGACT and R 5′ TGACTCCAACGATCAGCTGT; Flinn et al., 2013).

### Sample preparation and immunohistochemistry

All zebrafish (WT or *c9orf72^−/−^*) were anesthetized by immersing into 0.02% aminobenzoic acid ethyl-methyl-ester (Sigma: MS222) in PBS followed by intracardial perfusion with PBS to remove residual blood and 4% PFA for fixation. Bodies were incubated in 4% PFA overnight for postfixation. Spinal cord (SC) and skeletal muscle tissue were extracted using a stereo microscope. Skeletal muscle tissue was extracted from the right side of the dorsal part of the trunk, surrounding the spinal cord, starting caudal to the level of the operculum, until the level of the anterior border of the dorsal fin.

For 4c4 and Gfap immunofluorescent (IF) staining, SC was cryoprotected by incubation in 30% sucrose, o/n, 4°C, followed by OCT embedding and cryosectioning (15 μm). Sections were permeabilized with 0.25% Triton X-100, 10 min, followed by blocking with 5% animal serum, 0.25% Triton X-100 in PBS for 1 h, room temperature (RT). Subsequently, primary antibodies were applied in blocking solution overnight at 4°C. The next day, sections were washed and incubated with appropriate fluorescent secondary antibodies for 1 h, RT. Nuclei were counterstained with DAPI, followed by washing and mounting.

For choline acetyl transferase (ChAT) and neuromuscular junction (NMJ) staining, SC and skeletal muscle tissue was embedded in 4% agarose and cut into 50 μm or 150 μm thick sections, respectively. IF staining of vibratome sections was performed according to a protocol adapted from Kuscha et al. (2012). Vibratome sections were washed in 1× PBS-T (0.1% Triton X-100) for 10 min, RT; 2× 50 mM glycine in PBS-T for 10 min, RT, to remove residual PFA; and three times in PBS-T for 15 min, RT. Subsequently, sections were incubated in blocking solution (1% DMSO, 1% Donkey or Goat Serum, 1% BSA, 0.7% Triton X-100 in PBS) for 30 min, RT. Primary antibodies were applied overnight at 4°C in blocking solution. The following day, sections were washed and incubated with secondary antibodies in blocking solution overnight, 4°C. The third day, nuclei were counterstained with DAPI for 10 min, RT, followed by washing. *Z*-stacks of SC and muscle IF staining’s were acquired with the Zeiss Confocal 710.

For IF of ocular tissue, wild-type and mutant adult eyes were cryo-embedded in OCT prior to cryosectioning (18 µm thickness). Sections were rehydrated by incubating the slides in PBS (1×) for 5 min at RT. Antigen retrieval was performed by heating the slides in 10 mM sodium citrate, pH 6, for 20 min. Once at RT, slides were washed three times in 0.1% PBS-Triton X for 20 min, prior to incubation in blocking solution (1% BSA, 10% goat serum in 0.1% PBS-Triton-X) at RT for 1 h. Slides were then incubated with primary antibody solutions at 4°C overnight. The next day, excess antibodies were removed by washing slides three times in PBS for 20 min, followed by a 2 h incubation with secondary antibodies at RT. Alexa-488 goat anti-rabbit and Alexa-647 goat anti-mouse secondary antibodies used were from Invitrogen (1:1,000 dilution). Slides were washed three times in PBS for 20 min, the third wash containing DAPI stain (1:1,000). Finally, slides were mounted with coverslips and dried in the dark overnight. *Z*-stack images in the central retina were acquired on the Zeiss 900 Airyscan 2 confocal microscope, using the 40× objective lens.

Information about the antibodies and dilutions used is summarized in Table 1.

**Table 1.** Antibodies

Antibody	Cat code	Company	Dilution	Species
Glial fibrillary acidic protein (Gfap)	Z0334	Dako	1:500	Rabbit
4c4	–	Generated in house	1:100	Mouse
Choline acetyl transferase (ChAT)	AB144P	Sigma-Aldrich	1:100	Goat
Synaptic vesicle glycoprotein 2A (SV2A)	SV2	DSHB	1:100	Mouse (IgG1)
Donkey anti mouse IgG (H + L) Cy3	715-165-150	Jackson	1:200	Donkey
Donkey anti rabbit 647	A31573	ThermoFisher	1:1,000	Donkey
Donkey anti goat 555	A21432	ThermoFisher	1:500	Donkey
Goat anti mouse IgG1 488	A21121	ThermoFisher	1:1,000	Goat
Bungarotoxin	B35451	ThermoFisher	1:1,000	–
ZRF-1 (Gfap)	AB_10013806	ZIRC	1:200	Mouse
Ribeye A		Gift from T. Nicholson	1:2,000	Rabbit
GNAT2	PM075	MBL	1:100	Rabbit
PKC-α	Sc-208	Santa Cruz	1:300	Rabbit
HuC/D	A21271	Invitrogen	1:300	Mouse
GS	66323-1-Ig	Proteintech	1:200	Mouse
ZPR-3	AB_10013805	ZIRC	1:200	Mouse
ZPR-1	AB_10013803	ZIRC	1:200	Mouse
Rhodopsin (Rho)	n/a	Gift from David Hyde	1:2,000	Rabbit
Zo-1	33-9100	Invitrogen	1:100	Mouse
Goat anti rabbit-Alexa Fluor 488	A11008	Invitrogen	1:1,000	Goat
Goat anti mouse-Alexa Fluor 647	A21235	Invitrogen	1:1,000	Goat

### Image analysis

Maximum projections of *Z*-stacks were thresholded, converted into binary mask, and measured in Fiji/ImageJ. Threshold (4c4, Gfap, BF) was chosen manually for each batch which was processed simultaneously for WT and *c9orf72^−/−^* fish. Microglia and astrocyte area measurements were calculated as percentage of area post-thresholding normalized to BF area of the whole spinal cord section. Microglia, ChAT, and NMJ counts were counted manually per section. All image processing was performed blinded. Statistical analysis was performed as unpaired, parametric two-tailed *t* test.

All retinal quantifications were performed using Fiji/Image J on 3D *Z*-stacks. Neuronal numbers were determined by manually counting cells positive for both the neuronal markers of interest and DAPI, within a 100 × 100 × 10 mm ROI using the “multi-point” tool. 4c4^+^ microglia were counted in the entire field of view and in individual retinal layers. Retinal thickness measurements were carried out in DAPI-stained sections, and individual layer measurements were normalized to the overall retinal thickness from the top of the INL to the bottom of the RGC layer and expressed as a proportion of overall retinal thickness. Following Shapiro–Wilk normality tests to confirm normal distribution, statistical significance was determined using unpaired two-tailed *t* tests when comparing a single measurement between two groups. Two-way ANOVA was used when comparing multiple measurements within one image, for example, different layers in the same retina, or when comparing the same measurement between timepoints. Gfap immunofluorescence distribution along the apicobasal axis was quantified using IMARIS. Briefly, Gfap staining was segmented by generating a surface on IMARIS and dividing into the apical and basal halves. The volume was quantified automatically on IMARIS for the apical and basal regions, and a ratio between the two was quantified to calculate the apicobasal distribution in WTS and mutants. The number of microglia with ramified and ameboid morphology was quantified as previously described (Larbalestier et al., 2022).

## Results

### Mutant generation and validation of the *c9orf72^−/−^* mutant zebrafish line

**Figure 1.**
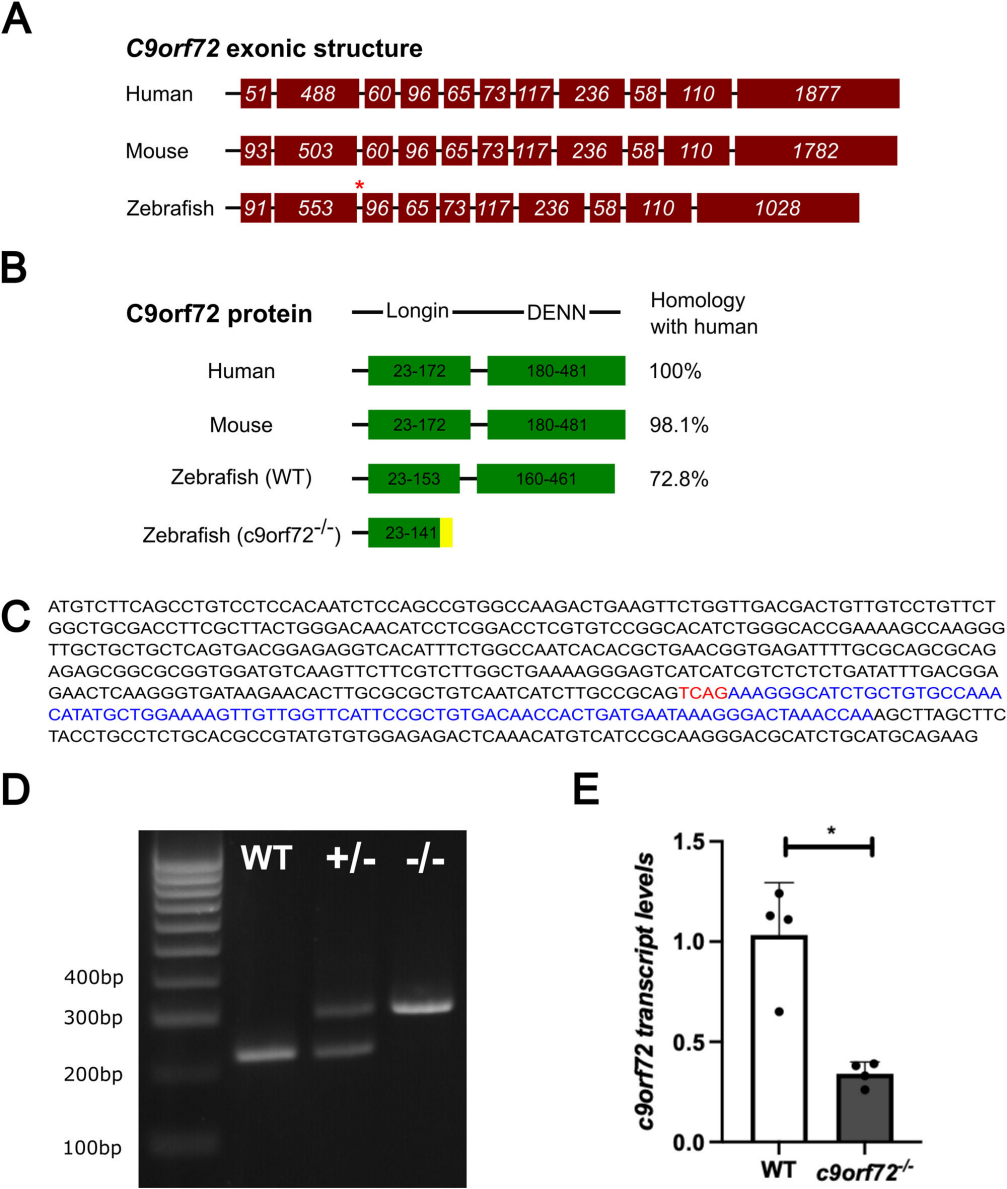
Characterization of the *c9orf72* loss of function mutation. ***A***, A comparison of exonic structure of human, mouse, and zebrafish *c9orf72* gene, with the number of base pairs in each exon denoted in white script. The absence of human/mouse exon 3 from the zebrafish gene is indicated by a red asterisk (see Extended Data Fig. 1-1). ***B***, C9orf72 amino acid (aa) sequence comparison/predicted protein domains of human, mouse, WT, and mutant zebrafish. The login domain of the WT zebrafish c9orf72 is shortened by 20 aa due to the absence of an exon which is present in humans/mice (exon 3). The mutant *c9orf72* zebrafish DNA sequence contains a frameshift mutation in exon 2, generated through CRISPR/Cas9. This results in a premature stop codon upon translation within the login domain, deleting the majority of zebrafish c9orf72 protein. The amino acid sequence after frameshift and prior to the premature stop codon is highlighted in yellow. ***C***, Exon 2 of *c9orf72* illustrating the frameshift mutation generated through CRISPR/Cas9. The frameshift allele is composed of a 4 bp deletion (red script) and 90 bp insertion (blue script) within WT exon 2 (black script). ***D***, Example genotyping gel demonstrating the large indel mutation visible by gel electrophoresis. ***E***, The mutation leads to a large 66% reduction in *c9orf72* transcript levels in the *c9orf72^−/−^* (*p* = 0.011; *n* = 4; Welch’s two-tailed *t* test), demonstrating activation of nonsense mediated decay by the frameshift allele, indicative of loss of function.

A single *C9ORF72* ortholog was identified in zebrafish (ENSDARG00000011837) using ENSEMBL. The zebrafish ortholog possesses ∼73% protein homology to the human protein as well as conserved gene synteny (Fig. 1*A*, Extended Data Fig. 1-1). Of note, the *C9ORF72* exon/intron structure was similar between zebrafish, mouse, and humans, with the exception of exon 3 (human/mouse), which was absent from the zebrafish *c9orf72* genomic sequence (Fig. 1*A*,*B*). To further understand the consequences of *c9orf72* deficiency in vivo, we generated a loss of function zebrafish line by targeting exon 2 using CRISPR/Cas9 (Fig. 1*A*). The selected *c9orf72* mutant allele contained a combined 4 bp deletion and 90 bp insertion, forming a frameshift and the generation of a premature stop coding within exon 2 (Fig. 1*A–C*). Due to the lack of reliable antibodies for *c9orf72* in zebrafish, we measured *c9orf72* transcript levels in the homozygous mutants at 5 days-post-fertilization (5 dpf). This revealed a 66% reduction of *c9orf72* mRNA (Fig. 1*D*; *p* = 0.011; *n* = 4; Welch’s two-tailed *t* test) in the *c9orf72^−/−^* compared with WTS controls demonstrating the frameshift allele causes nonsense mediated decay, indicating the mutation is loss of function.

10.1523/JNEUROSCI.2128-23.2024.f1-1 Figure 1-1 **Schematic of conserved gene synteny between the human *C9ORF72* and zebrafish *c9orf72*.** In both species the genes encoding C9ORF72 and LINGO2 are located within 0.5mb of each other on the same chromosome. Download Figure 1-1, TIF file.

### Absence of neuronal loss and NMJ degeneration in the spinal cord of *c9orf72^−/−^*

ALS is characterized by the loss of MNs, leading to muscle weakness and ultimately death due to respiratory failure (Hardiman et al., 2017). Crucially, the NMJ, the synaptic connection between MNs and skeletal muscle, is subject to degeneration early during ALS pathology which precedes MN loss. As such, we were interested in understanding the effect of *C9ORF72* deficiency on spinal cord MNs and NMJs in adults at an advanced stage of their life course. Zebrafish are known to have an average lifespan up to 42 months (Gerhard et al., 2002), and age-related neurodegeneration begins to occur at ∼24 months (Yu et al., 2006; Martins et al., 2022), as such we selected 24 months postfertilization (mpf) for analysis in *c9orf72^−/−^* mutants.

**Figure 2.**
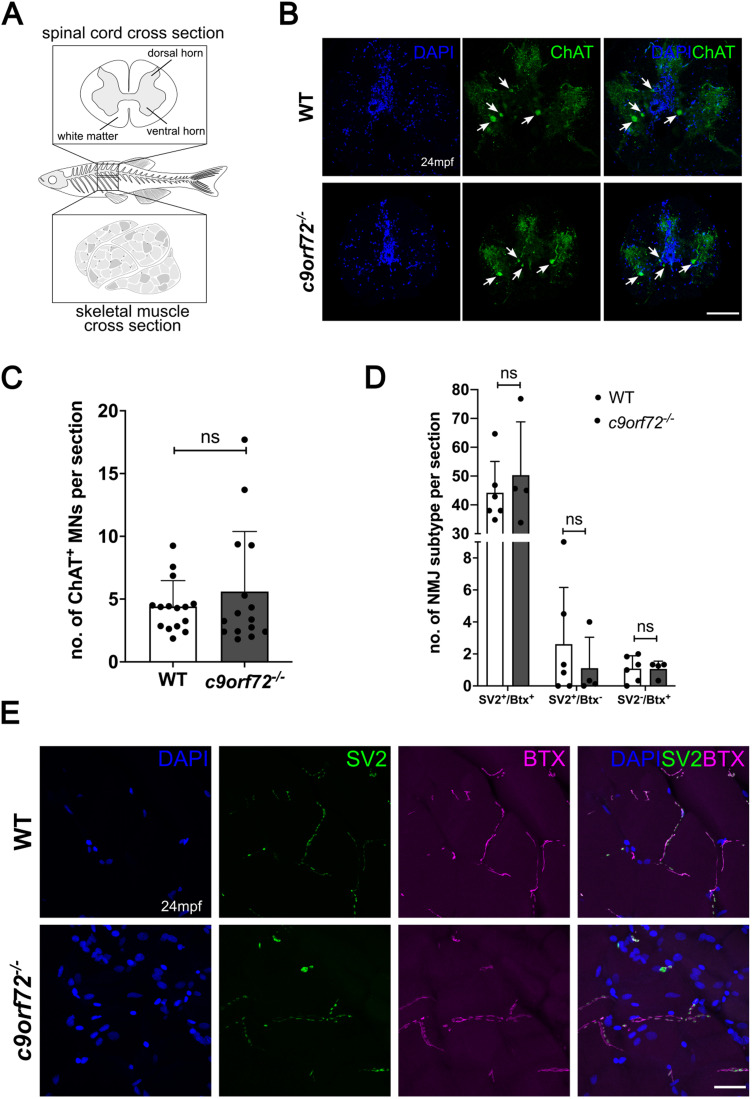
*c9orf72* deficiency does not lead to MN loss or NMJ degeneration in adult zebrafish spinal cord. ***A***, Schematic diagram of adult zebrafish, highlighting the spinal cord region used for analysis. ***B***, Representative immunofluorescent image of *ChAT* staining in WT and *c9orf72*^−/−^ spinal cord of 24-month-old zebrafish. Scale bar, 100 μm. ***C***, Quantification of ***A***. MN numbers were not significantly different between WT (2.92 ChAT^+^ MNs per section) and *c9orf72^−/−^*(3.37 ChAT^+^ MNs per section) zebrafish. Unpaired, two-tailed, and parametric *t* test, *p* = 0.38, *n* = 15 per genotype. SV2, presynaptic marker; BTX, postsynaptic marker labeling AChRs. Scale bar, 25 μm. ***D***, Quantification of ***E***. NMJ integrity was not altered in *c9orf72*^−/−^ zebrafish. SV2^+^/BTX^+^: WTS, 44.19, *c9orf72^−/−^*, 50.32, *p* = 0.52; SV2^+^/BTX^−^: WTS, 2.61, *c9orf72^−/−^*, 1.12, *p* = 0.47; SV2^−^/BTX^+^: WTS, 1.08, *c9orf72^−/−^*, 1.06, *p* = 0.96. Multiple unpaired, two-tailed, and parametric *t* test, *n*-WTS:6, *n*- *c9orf72^−/−^*:4. ***E***, Representative immunofluorescent image of NMJ staining in WT and *c9orf72^−/−^* muscle of 24-month-old zebrafish. Scale bar, 25 μm.

Firstly, we stained for the mature cholinergic MN marker ChAT. No difference in ChAT^+^ MN numbers was observed between WTS (2.92 ChAT^+^ MNs per section) and *c9orf72*^−/−^ (3.37 ChAT^+^ MNs per section; *p* = 0.38; *n* = 15; unpaired, parametric *t* test), highlighting no MN loss (Fig. 2*A–C*). NMJ degeneration precedes MN loss in ALS mouse models as well as clinical studies (Walker et al., 2015; Bashford et al., 2020; Altman et al., 2021); therefore, we analyzed NMJ structure within our *c9orf72^−/−^* to potentially detect subtle changes toward MN health. Skeletal muscle was stained for synaptic vesicle 2 (SV2, presynaptic marker) and acetylcholine receptor [AChR, postsynaptic marker, labeled with bungarotoxin (BTX); Fig. 2*D–E*]. We distinguished between SV2^+^/BTX^+^ (innervated NMJ; WTS, 44.19; *c9orf72^−/−^*, 50.32; *p* = 0.52; unpaired, parametric *t* test), SV2^+^/BTX^−^ (presynapse only; WTS, 2.61; c9orf72^−/−^, 1.12; *p* = 0.47; *n* = 4–6; unpaired, parametric *t* test), and SV2^−^/BTX^+^ (postsynapse only; WTS, 1.08; c9orf72^−/− ^=^ ^1.06; *p* = 0.96; *n* = 4–6; unpaired, parametric *t* test; Fig. 2*D–E*). However, no difference in any of the NMJ subtypes was observed (Fig. 2*D–E*). Taken together, *c9orf72* deficiency in adult zebrafish does not lead to spinal cord MN loss or NMJ degeneration at 24 months of age.

### Lack of gliosis in the spinal cord *c9orf72^−/−^* mutants

**Figure 3.**
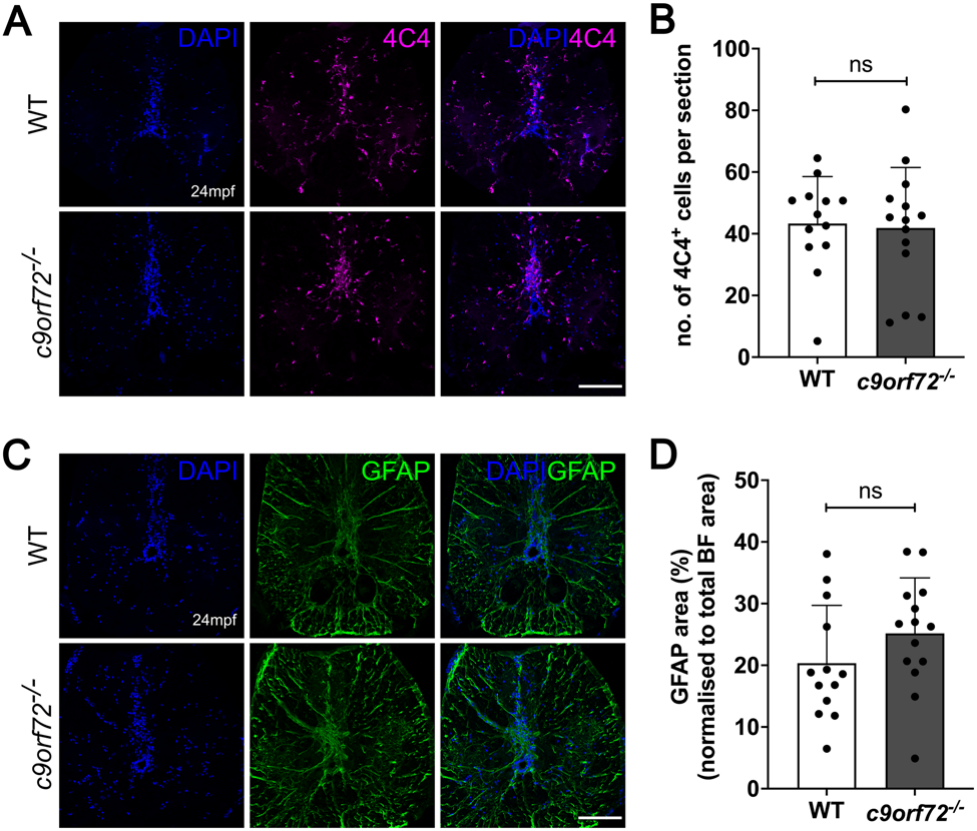
*c9orf72*^−/−^ adult zebrafish do not exhibit micro- or astrogliosis in the spinal cord. ***A***, Representative immunofluorescent image of 4c4^+^ microglia in WTS and *c9orf72*^−/−^ spinal cord of 24-month-old zebrafish. Scale bar, 100 µm. ***B***, Quantification of ***A***. Microglial numbers per spinal cord section are unaltered between WTS (43.32 4c4^+^ microglia) and *c9orf72^−/−^* (41.85 4c4^+^ microglia). Unpaired, two-tailed, and parametric *t* test; *p* = 0.83; *n*-WTS: 13; *n*-*c9orf72^−/−^*, 14. See Extended Data Figures 3-1 and 3-2. ***C***, Representative immunofluorescent image of Gfap staining in WTS and *c9orf72*^−/−^ spinal cord of 24-month-old zebrafish. Scale bar, 100 µm. ***D***, Quantification of ***C***. Gfap^+^ area within spinal cord, normalized to spinal cord size, does not differ between WTS (20.34%) and *c9orf72^−/−^* (25.18%). Unpaired, two-tailed, and parametric *t* test; *p* = 0.18; *n*-WTS, 13; *n*-*c9orf72^−/−^*, 14.

Consistent with our findings, *C9ORF72* KO mouse models also do not exhibit MN loss or NMJ degeneration but do develop an increased neuroinflammatory signature, including microgliosis (O’Rourke et al., 2016; Lall et al., 2021). Therefore, we were interested in understanding if glial activation was present in the CNS of our *c9orf72^−/−^* zebrafish. Spinal cord sections were stained for the zebrafish specific microglial marker 4c4 (Becker et al., 1998) and glial fibrillary acidic protein (Gfap), labeling astrocytes and radial glia. Microglial number was not significantly altered in the spinal cord of *c9orf72^−/−^* zebrafish compared with WTS (*p* = 0.83; *n* = 13–14; Fig. 3*A*,*B*; unpaired, parametric *t* test) and did not show obvious morphological signs of microglial activation (i.e., ramified vs spheroid) in *c9orf72^−/−^* (Extended Data Fig. 3-1). Furthermore, microglial area was also not notably increased in *c9orf72^−/−^* (WTS, 2.9%; *c9orf72^−/−^*, 3.37%; *p* = 0.50; *n* = 13–14; unpaired, parametric *t* test; data not shown). Equally, Gfap^+^ area within the spinal cord was not significantly altered comparing WTS (20.34%) and *c9orf72*^−/−^ (25.18%; *p* = 0.18; *n* = 13–14; Fig. 3*C*,*D*; unpaired, parametric *t* test). Overall, these results suggest the absence of neuroinflammation in adult *c9orf72^−/−^* zebrafish spinal cord. To identify potentially more subtle neuroinflammatory signatures within the CNS, we analyzed chitotriosidase activity levels (a marker of generalized neuroinflammation) in brain homogenates. No statistical differences were found between *c9orf72^−/−^* and WTS confirming a lack of inflammatory signatures in the *c9orf72^−/−^* (Extended Data Fig. 3-2). To ascertain whether *c9orf72* deficiency results in lysosomal dysfunction within the CNS, we analyzed the activity of the lysosomal enzymes, β-hexosaminidase and β-galactosidase, in brain homogenates. The activity of both enzymes was not significantly altered between genotypes (Extended Data Fig. 3-2), suggesting lysosomal function is not grossly altered in the brains of our *c9orf72^−/−^*.

10.1523/JNEUROSCI.2128-23.2024.f3-1 Figure 3-1 **Morphology of spinal cord microglia is not altered in *c9orf72^-/-^*.** A) Example images of amoeboid and ramified spinal cord microglia at 24mpf. Scale bar: 10  mm. B) Quantification of Figure 3A, taking into account the morphological differences of amoeboid (WT: 14.57, *c9orf72^-/-^*: 12.24) and ramified (WT: 38.49, *c9orf72^-/-^*: 29.62) microglia, which is unaltered in *c9orf72^-/-^* mutants. Unpaired, two-tailed and parametric t-test, amoeboid: p = 0.5765, n-WT:13, n- *c9orf72^-/-^*:14, ramified: p = 0.3087, n-WT:13, n- *c9orf72^-/-^*:14. Download Figure 3-1, TIF file.

10.1523/JNEUROSCI.2128-23.2024.f3-2 Figure 3-2 **Lysosomal enzyme activities are not significantly altered between *c9orf72^-/-^* brains and WT controls**. Measurements of different enzyme activities from whole brain homogenates revealed no statistical difference in activity between genotypes. These included Chitotriosidase activity (2A, p = 0.6704) a general marker of neuroinflammation and potential ALS biomarker. The lysosomal enzyme enriched in microglia, b Hexosaminidase (2B, p = 0.5156) and the lysosomal enzyme b Galactosidase (2C, p = 0.5188). Unpaired, two-tailed and parametric t-test, n = 4 for all. These data indicate there is minimal neuroinflammation occurring in the brain. Download Figure 3-2, TIF file.

### *c9orf72*^−/−^ mutants show signs of neurodegeneration in the retina

**Figure 4.**
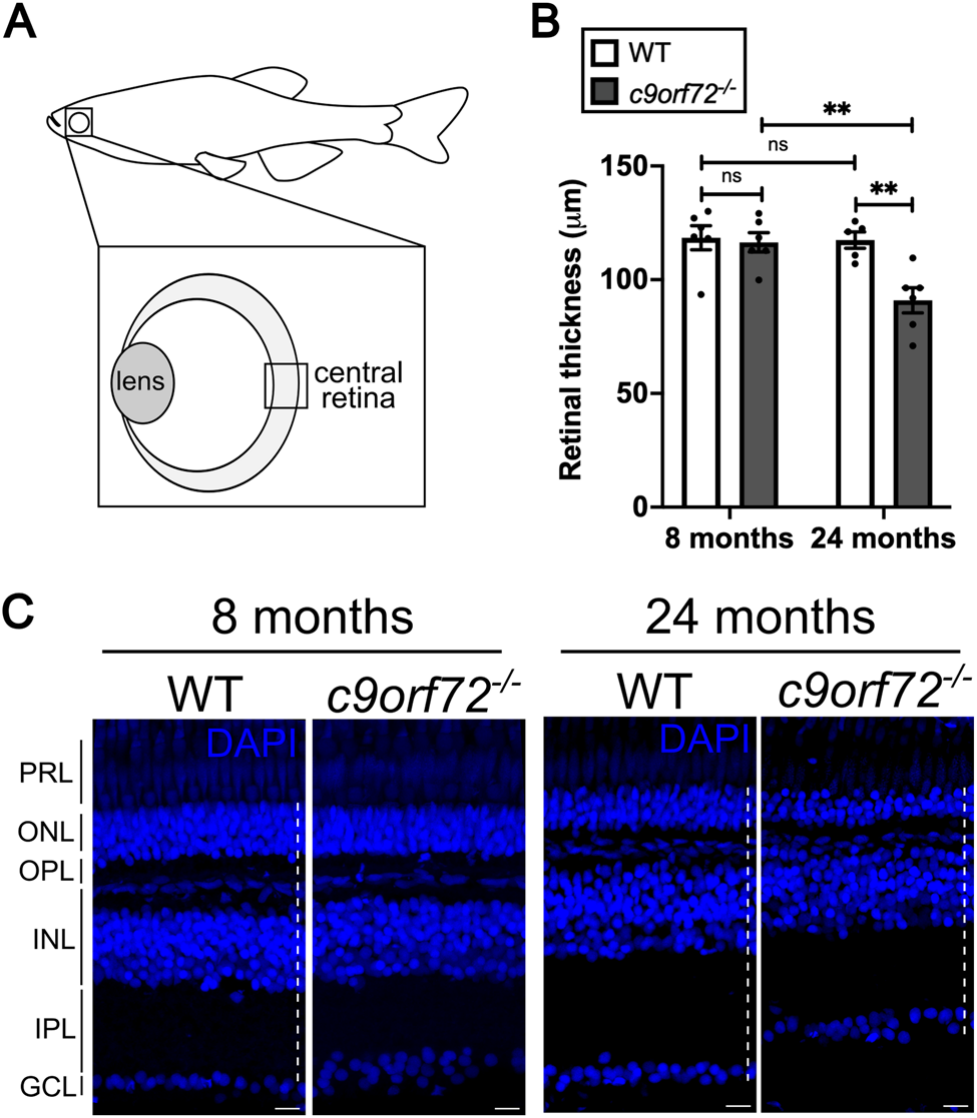
*c9orf72^−/−^* mutants exhibit signs of degeneration of inner retinal neurons. ***A***, Schematic diagram of adult zebrafish retina, highlighting the central retinal region used for analysis. ***B***, Quantification of mean retinal thickness of WTS and *c9orf72^−/−^* at 8 and 24 mpf, two-way ANOVA, Šídák’s multiple-comparisons test; 8 mpf WTS versus 8 mpf *c9orf72^−/−^*: *p* = 0.9905; 8 mpf WTS versus 24 mpf WTS *p* = 0.9988; 24 mpf WTS versus 24 mpf *c9orf72^−/−^*: *p* = 0.0062; 8 mpf *c9orf72^−/−^* versus 24 mpf *c9orf72^−/−^*: *p* = 0.0062. ***C***, DAPI (blue) staining showing the nuclear layers of the WTS and *c9orf72^−/−^* retina at 8 and 24 mpf. In the innermost retina, retinal ganglion cell (RGC) somata make up the ganglion cell layer (GCL) and project processes into the inner plexiform layer, where they form synapses with bipolar and amacrine cells, the cell bodies of which are found in the inner nuclear layer (INL). Apical to the INL is the outer plexiform layer (OPL), where bipolar cells and horizontal cells connect to the photoreceptors in the outer nuclear layer (ONL). In the outermost retina, the inner and outer segments of rod and cone photoreceptors make up the photoreceptor layer (PRL). Dotted line represents the approach to measure retinal thickness. *n* = 5–6 retinas per genotype. Scale bar, 50 µm. See also Extended Data Figures 4-1–4-4).

Ocular defects in *C9ORF72*-associated disease have been described in patients (Moss et al., 2012; Fawzi et al., 2014), as well as in *Drosophila* models of c9orf72-associated ALS (Sharpe et al., 2021). However, how C9ORF72 deficiency impacts the retina has yet to be described in a vertebrate model system. The retina is a highly structured and conserved CNS tissue, organized into distinct nuclear layers, separated by synaptic plexiform layers. Here we focused on the central retina (Fig. 4*A*), as it has been shown to display retinal degeneration earlier than peripheral regions in the aging WTS zebrafish (Van houcke et al., 2019; Martins et al., 2022). As thinning of the retina is a hallmark sign of neurodegeneration, we assessed overall tissue thickness in sections from WTS and *c9orf72*^−/−^ mutant retinas. At 5 dpf, retinal thickness and neuronal/glial counts are unaffected in *c9orf72^−/−^*, suggesting development is not perturbed (Extended Data Figs. 4-1–4-3). At the adult timepoint of 8 mpf, the mean retinal thickness of c*9orf72^−/^*^−^ mutant retinas did not differ from WTS [Fig. 4*B*,*C*; *p* = 0.9905; two-way ANOVA; Šídák’s multiple-comparisons test (Šídák’s ANOVA); *n* = 6] but was ∼20% thinner than WTS at 24 mpf (*p* = 0.0067; Šídák’s ANOVA; *n* = 5–6). To understand whether the reduction in overall retinal thickness observed in the c9*orf72^−/−^* mutants is a consequence of the thinning of one particular layer, we next measured the ratios of each layer of the retina and compared them between genotypes. Although the overall retinal thickness was comparable between mutants and WTS at 8 mpf, measurements of the ratios of the individual layers revealed a decreased ratio of the INL in *c9orf72^−/−^*, suggesting that this nuclear layer may begin thinning first (Extended Data Fig. 4-4). We did not observe any significant differences between the ratios of individual layers in WTS and *c9orf72^−/−^* retinas at 24 mpf (Extended Data Fig. 4-4), suggesting that all layers are thinning to a similar extent and equally contributing to the overall reduction in retinal thickness in the aged retinas.

10.1523/JNEUROSCI.2128-23.2024.f4-1 Figure 4-1 **Inner retinal development is normal in *c9orf72^-/-^* mutants.** A) Schematic diagram of 5-day post fertilisation (dpf) zebrafish retina, highlighting the central retinal region used for analysis. B) DAPI (blue) staining showing the nuclear layers of the WT and *c9orf72^-/-^* larval retina and overall central retinal thickness. C) Quantification of mean thickness of WT and *c9orf72^-/-^* retinas; unpaired t-test; p = 0.0704. D) Antibody staining for bipolar cell marker, (PKC-b, green), amacrine and retinal ganglion cells (HuC/D, magenta) and nuclei stained with DAPI (blue) in WT and *c9orf72^-/-^* retinas. E) Quantification of number of HuC/D^+^ amacrine cells in INL per 100  mm x 100 × 10  mm ROI; unpaired t-test; p = 0.0726. F) Quantification of number of HuC/D^+^ retinal ganglion cells in GCL per 100  mm x 100 x10  mm ROI; unpaired t-test; p = 0.7723. G) Quantification of number of PKC-b^+^ bipolar cells in INL per 100  mm x 100 x10  mm ROI; unpaired t-test; p = 0.7024; n = 11-12 larvae per genotype; Scale bars, 10  mm. Download Figure 4-1, TIF file.

10.1523/JNEUROSCI.2128-23.2024.f4-2 Figure 4-2 **Rod and cone photoreceptors do not exhibit developmental defects in *c9orf72^-/-^ mutants*.** A) Immunostaining for pan-cone marker, Gnat2 (green) and double cone marker, Zpr-1 (magenta) in WT and c9orf72-deficient retinal cryosections at 5dpf. Nuclei labelled with DAPI (blue). B) Quantification of mean number of Gnat2-positive cone photoreceptors in WT and *c9orf72*-deficient retinas;100  mm x 100 × 10  mm ROI; unpaired t-test, p = 0.8238. C) Quantification of mean number of Zpr-1-positive cone photoreceptors in WT and *c9orf72*-deficient retinas;100  mm x 100 × 10  mm ROI; unpaired t-test, p = 0.8209. D) Antibody staining for Rhodopsin (Rho; green) and rod/double cone marker Zpr-3 (magenta). E) Close up of individual Rho + Zpr-3-expressing rods in WT and mutants. F) Quantification of mean number of Rho + Zpr-3^+^ rod photoreceptors in WT and c9orf72-deficient retinas;100  mm x 100  mm ROI; unpaired t-test, p < 0.0001. n = 10-12 retinas per genotype. Scale bars, 10  mm. Download Figure 4-2, TIF file.

10.1523/JNEUROSCI.2128-23.2024.f4-3 Figure 4-3 **Glial development is unaffected in *c9orf72^-/-^* deficient retinas.** A) Immunostaining for gliosis marker, Gfap (magenta) in WT and *c9orf72*-deficient retinal cryosections at 5dpf. Nuclei labelled with DAPI (blue). B) Ratio of mean Gfap distribution in apical versus basal regions of the retina; unpaired t-test, p = 0.7791. C) Antibody labelling of retinal microglia with 4c4 (magenta), cell bodies labelled with DAPI (blue). D) Higher magnification image of 4c4 and DAPI staining. E) Quantification of the average number of 4c4^+^ microglia per image; unpaired t-test; p = 0.6226; Scale bars, 10  mm. Download Figure 4-3, TIF file.

10.1523/JNEUROSCI.2128-23.2024.f4-4 Figure 4-4 **Retinal layer become proportionally thinner in *c9orf72^-/-^* mutants with age.** A) Quantification of the thickness ratios of retinal layers compared to the overall retinal thickness in WTS and *c9orf72^-/-^* mutants at 8months post fertilisation (mpf). Ganglion cell layer (GCL): p = 0.9995, inner plexiform layer (IPL): p = 0.1333, inner nuclear layer (INL): p = 0.0025, outer plexiform layer (OPL): p = 0.9990 and outer nuclear layer (ONL): p = 0.9994. Two-way ANOVA, Šídák's multiple comparisons test; n = 6 fish per genotype. B) Quantification of the thickness of each retinal layers compared to the overall retinal thickness in WTS and *c9orf72^-/-^* mutants at 24mpf. GCL: p = 0.9172, IPL: p = 0,3725 INL:p > 0.9999, OPL: p = 0.9398 and ONL:p = 0.5942. Two-way ANOVA, Šídák's multiple comparisons test; n = 5-6 fish per genotype. Download Figure 4-4, TIF file.

**Figure 5.**
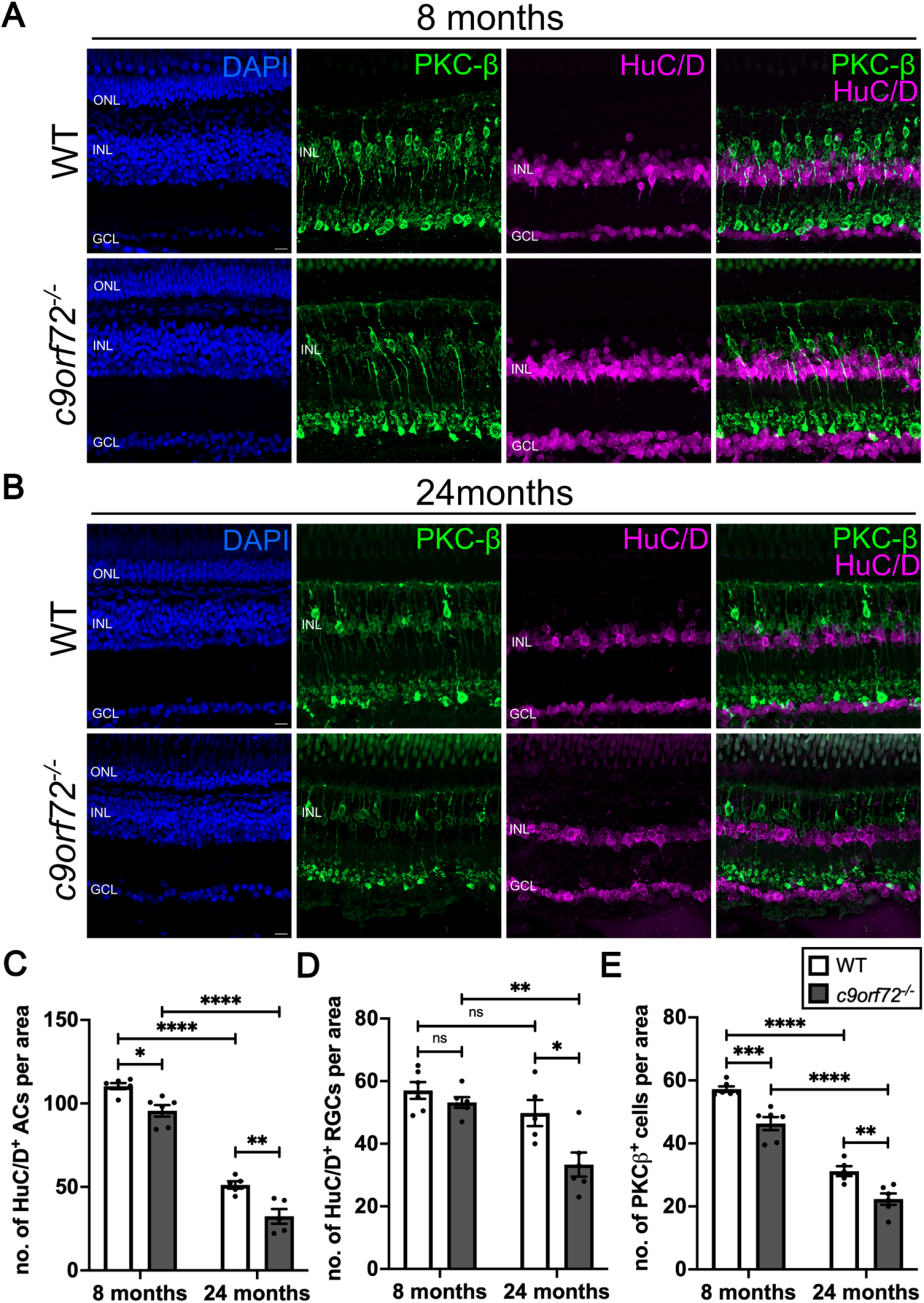
*c9orf72^−/−^* mutants exhibit signs of degeneration of inner retinal neurons. ***A***, Antibody staining for bipolar cell marker (PKC-β, green), amacrine and retinal ganglion cells (HuC/D, magenta) and nuclei (DAPI, blue) in WTS and *c9orf72^−/−^* retinas at 8 mpf and (***B***) at 24 mpf. ***C***, Quantification of number of HuC/D^+^ amacrine cells (ACs) in the inner nuclear layer (INL) per 100 µm × 100 µm ROI in WTS and *c9orf72^−/−^* mutants at 8 and 24 mpf; two-way ANOVA, Šídák’s multiple-comparisons test; 8 mpf WTS versus 8 mpf *c9orf72^−/−^*: *p* = 0.0120; 8 mpf WTS versus 24 mpf WTS *p* < 0.0001; 24 mpf WTS versus 24 mpf *c9orf72^−/−^*: *p* = 0.0034; 8 mpf *c9orf72^−/−^* versus 24 mpf *c9orf72^−/−^*: *p* < 0.0001. ***D***, Quantification of number of HuC/D^+^ retinal ganglion cells (RGCs) in ganglion cell layer (GCL) per 100 µm × 100 µm ROI in WTS and *c9orf72^−/−^* mutants at 8 and 24 mpf; two-way ANOVA, Šídák’s multiple-comparisons test; 8 mpf WTS versus 8 mpf *c9orf72^−/−^*: *p* = 0.9502; 8 mpf WTS versus 24 mpf WTS *p* = 0.5792; 24 mpf WTS versus 24 mpf *c9orf72^−/−^*: *p* = 0.0120; 8 mpf *c9orf72^−/−^* versus 24 mpf *c9orf72^−/−^*: *p* = 0.0014. ***E***, Quantification of number of PKC-β ^+ ^bipolar cells (BPCs) in the inner nuclear layer (INL) per 100 µm × 100 µm ROI in WTS and *c9orf72^−/−^* mutants at 8 and 24 mpf; two-way ANOVA, Šídák’s multiple-comparisons test; 8 mpf WTS versus 8 mpf *c9orf72^−/−^*: *p* = 0.0008; 8 mpf WTS versus 24 mpf WTS *p* < 0.0001; 24 mpf WTS versus 24 mpf *c9orf72^−/−^*: *p* = 0.0095; 8 mpf *c9orf72^−/−^* versus 24 mpf *c9orf72^−/−^*: *p* < 0.0001. *n* = 5–6 retinas per genotype. Scale bars, 50 µm.

To determine whether the observed decrease in retinal thickness was due to the loss of a specific neuronal cell type, we performed immunostaining for known neuronal markers of the inner retina in WTS and *c9orf72^−/−^*. Antibody staining for HuC/D, a marker for amacrine (AC) and retinal ganglion cells (RGCs), revealed a small reduction in AC numbers by 13% in *c9orf72^−/−^* 8 mpf compared with WTS (Fig. 5*A*,*C*; *p* = 0.0120; Šídák’s ANOVA; *n* = 6) which increased to 36.8% by 24 mpf (Fig. 5*B*,*D*; *p* = 0.0034; Šídák’s ANOVA; *n* = 5–6). HuC/D + RGCs were not significantly different between *c9orf72^−/−^* and WTS at 8 mpf (Fig. 5*A*,*D*; *p* = 0.9502; Šídák’s ANOVA; *n* = 6). However, by 24 mpf we observed a 33% reduction in RGC number (*p* = 0.0120; Šídák’s ANOVA; *n* = 5–6). Furthermore, we observed a reduction in the number of PKC-β-positive bipolar cells (BPCs), another type of retinal interneuron, between WTS and *c9orf72^−/−^* at both 8 and 24 mpf (Fig. 5*A*,*B*,*F*), which exhibited decreases compared with WTS of 10% (*p* = 0.0008; Šídák’s ANOVA; *n* = 6) and 28% (*p* = 0.0095; Šídák’s ANOVA; *n* = 5–6), respectively. This early reduction in INL interneurons correlates with the apparent thinning of the INL observed at 8 mpf (Extended Data Fig. 4-4). It should be noted that we also observed a significant reduction in all cell types in WTS over time due to aging; however, this was exacerbated in the *c9orf72^−/−^* mutants.

Together, these data identify progressive retinal degeneration in *c9orf72^−/−^* mutants with age that appears to, at least in part, be attributed to an initial loss of neurons in the INL (inner nuclear layer, ∼30% of each cell type).

### *c9orf72*^−/−^ mutants exhibit signs of photoreceptor degeneration

**Figure 6.**
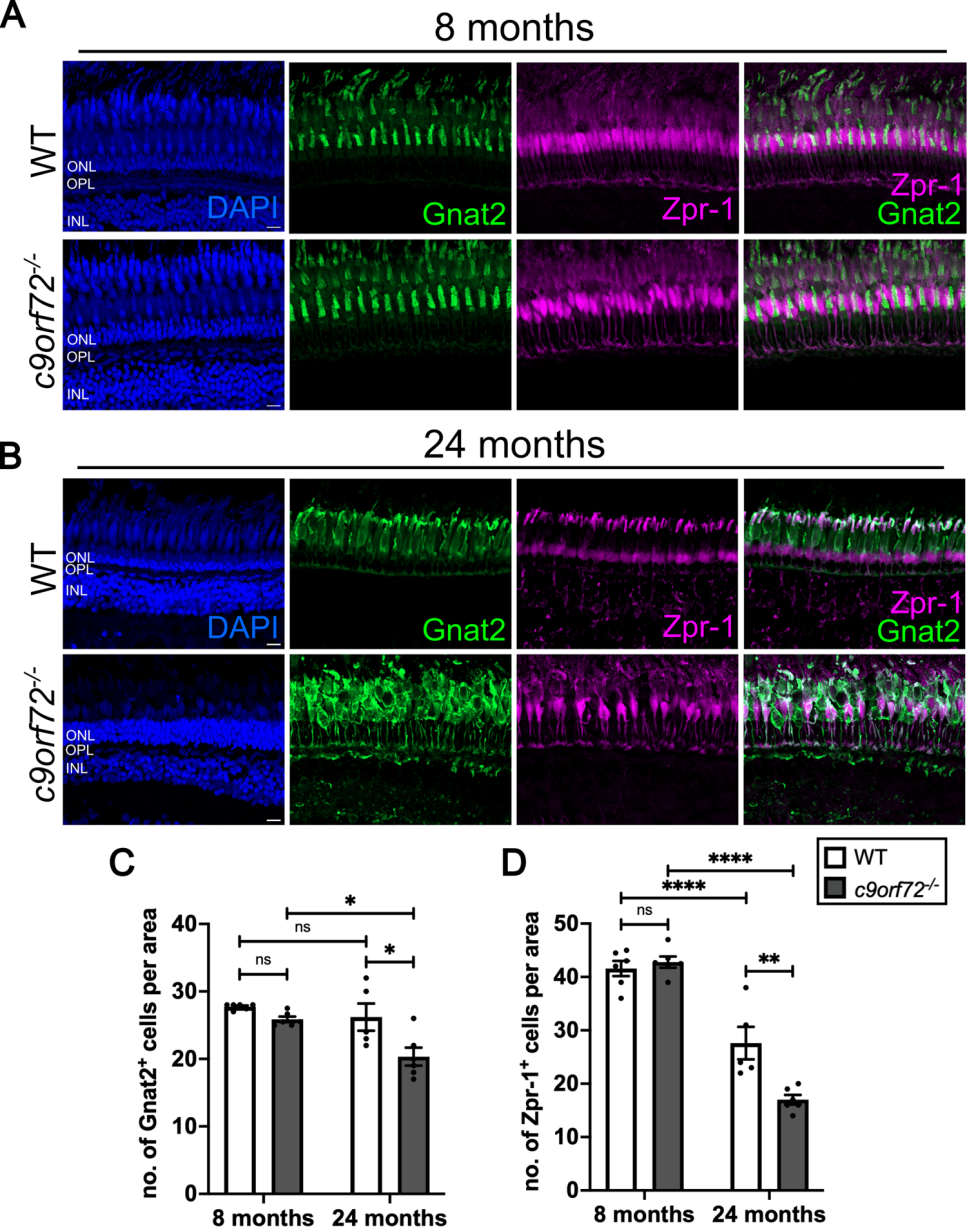
Cone photoreceptor degeneration in *c9orf72^−/−^* mutants. ***A***, Immunostaining for pan-cone marker, Gnat2 (green) and double cone marker, Zpr-1 (magenta) in WTS and ***B ***c9orf72-deficient retinal cryosections. Nuclei labeled with DAPI (blue). ***C***, Quantification of mean number of Gnat2-positive cone photoreceptors in WTS and *c9orf72*-deficient retinas at 8 and 24 mpf; 100 µm × 100 µm × 10 µm ROI; two-way ANOVA, Šídák’s multiple-comparisons test, 8 mpf WTS versus 8 mpf *c9orf72^−/−^ p* = 0.06058, 8 mpf WTS versus 24 mpf WTS *p* = 0.7875; 8 mpf *c9orf72^−/−^* versus 24 mpf *c9orf72^−/−^ p* = 0.0128; 24 mpf WTS versus 24 mpf *c9orf72^−/−^ p* = 0.0104. ***D***, Quantification of mean number of Zpr**-**1-positive cone photoreceptors in WTS and *c9orf72*-deficient retinas at 8 and 24 mpf; 100 µm × 100 µm × 10 µm ROI; two-way ANOVA, Šídák’s multiple-comparisons test, 8 mpf WTS versus 8 mpf *c9orf72^−/−^ p* = 0.9537; 8 mpf WTS versus 24 mpf WTS *p* < 0.0001; 8 mpf *c9orf72^−/−^* versus 24 mpf *c9orf72^−/−^ p* < 0.0001; 24 mpf WTS versus 24 mpf *c9orf72^−/−^ p* = 0.0017; *n* = 5–6 fish per genotype. Scale bars, 50 µm.

Having identified signs of progressive neuronal loss of inner retinal neurons in *c9orf72^−/−^* mutants, we next examined the consequences of *c9orf72* deficiency on the outer retinal cell types by analyzing cone (Gnat2 and Zpr-1) and rod (Rho and Zpr-3) photoreceptor markers. Quantification of Gnat2-positive photoreceptors per area at 8 mpf did not reveal any significant differences between WTS and *c9orf72^−/−^* (Fig. 6*A*,*C*; *p* = 0.6058; Šídák’s ANOVA; *n* = 6). However, a ∼23% reduction in Gnat2-positive cone photoreceptors was detected in *c9orf72^−/−^* at 24 mpf (Fig. 6*B*,*C*; *p* = 0.0104; Šídák’s ANOVA; *n* = 5–6). In a similar fashion, antibody staining with Zpr-1 revealed a significant reduction in cone number between the genotypes only at 24 mpf (Fig. 6*D*; *p* = 0.0017; Šídák’s ANOVA; *n* = 5–6). In a similar manner to the inner retinal neurons, there was a significant drop in Zpr-1-positive cells between WTS at 8 and 24 mpf (Fig. 6*D*), suggesting age-related degeneration. However, this loss of cones was markedly higher in *c9orf72^−/−^* mutants than that in WTS.

**Figure 7.**
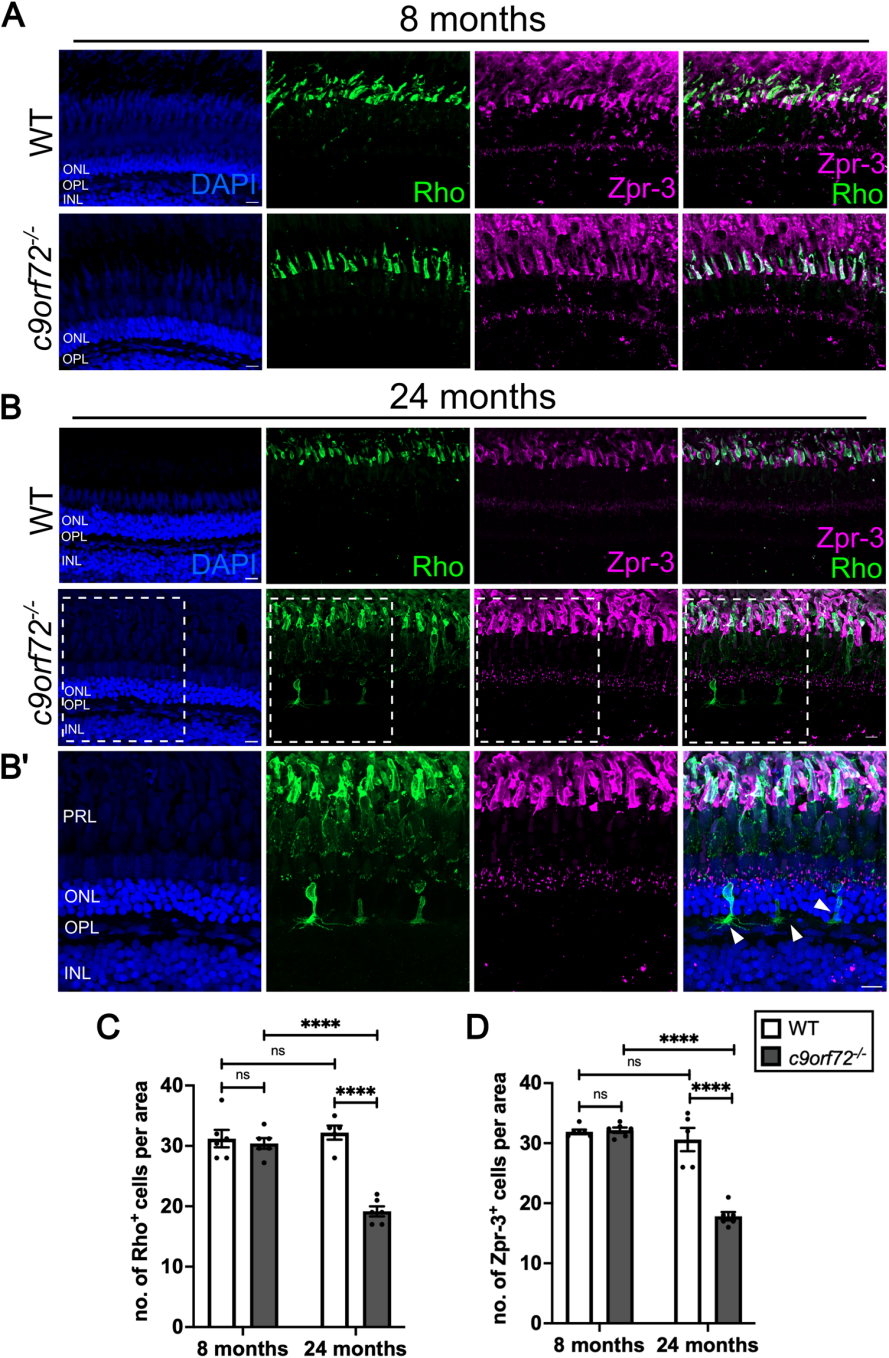
Rod photoreceptor degeneration in *c9orf72^−/−^* mutants. ***A***, Antibody staining for rod photoreceptor marker rhodopsin (Rho, green) and rod and green-cone photoreceptor marker (Zpr-3, magenta) and nuclei (DAPI, blue in WTS and *c9orf72^−/−^* retinas at 8 mpf and B at 24 mpf. ***B***’, Close up of *c9orf72^−/−^* retina from ***B*** with nuclei labeled with DAPI (blue). ***C***, Quantification of mean number of Rho-positive rod photoreceptors in WTS and *c9orf72*-deficient retinas at 8 and 24 mpf; 100 mm × 100 µm × 10 mm ROI; two-way ANOVA; 8 mpf WTS versus 8 mpf *c9orf72^−/−^ p* = 0.9525; 8 mpf WTS versus 24 mpf WTS *p* = 0.0233; 8 mpf *c9orf72^−/−^* versus 24 mpf *c9orf72^−/−^ p* < 0.0001; 24 mpf WTS versus 24 mpf *c9orf72^−/−^ p* < 0.0001. ***D***, Quantification of mean number of Zpr-3-positive rod and green cone photoreceptors in WTS and *c9orf72*-deficient retinas at 8 and 24 mpf;100 mm × 100 µm × 10 mm ROI; two-way ANOVA; 8 mpf WTS versus 8 mpf *c9orf72^−/−^ p* = 0.9970; 8 mpf WTS versus 24 mpf WTS *p* = 0.7859; 8 mpf *c9orf72^−/−^* versus 24 mpf *c9orf72^−/−^ p* < 0.0001; 24 mpf WTS versus 24 mpf *c9orf72^−/−^ p* < 0.0001. WTS; white arrowheads indicate displaced photoreceptors; phenotype observed in 0/5 WTS retinas and 5/6 *c9orf72^−/−^* retinas; *n* = 5–6 fish per genotype; PRL, photoreceptor layer; ONL, outer nuclear layer; OPL, outer plexiform layer; INL, inner nuclear layer. Scale bar, 10 µm.

Subsequently, we assessed the number of Rho- and Zpr-3-positive rod photoreceptors at the same adult stages. We detected no difference between WTS and *c9orf72^−/−^* at 8 mpf (Fig. 7*A*,*C*; *p* = 0.9525; Šídák’s ANOVA; *n* = 6) or between WTS at either adult timepoint (*p* = 0.9233; Šídák’s ANOVA; *n* = 5–6). However, at 24 mpf we saw a 40% decrease in the number of Rho-positive rods between WTS and *c9orf72^−/−^* mutants (Fig. 7*C*; *p* < 0.0001; Šídák’s ANOVA; *n* = 5–6).

Quantification of Zpr-3, a marker for rods and a subpopulation of cone photoreceptors, did not reveal a significant difference between WTS and *c9orf72^−/−^* at 8 mpf (Fig. 7*A–C*; *p* = 0.9970; Šídák’s ANOVA; *n* = 6). In contrast, at 24 mpf the mean number of Zpr-3-positive cells was 42% lower in *c9orf72^−/−^* mutants relative to WTS (Fig. 7*D*; *p* < 0.0001; Šídák’s ANOVA; *n* = 5–6). Strikingly, aside from the presence of fewer rods in the aged *c9orf72^−/−^* retina (Fig. 7*C*,*D*), we observed the mislocalization of rhodopsin to the cell bodies and processes in the OPL (outer plexiform layer), instead of being confined to the outer segments as observed in WTS (Fig. 7*B’*). Although the occurrence of this was sporadic, with only 1–3 cells per central retinal region analyzed, this phenomenon was exclusive to the *c9orf72^−/−^*, and not seen in any WTS. Altogether, our findings point to a global loss of photoreceptor types in the *c9orf72^−/−^* retina at 24 mpf, which was not observed at 8 mpf when interneuron loss is present.

### Gliosis and microglial redistribution accompany neuronal degeneration in the c*9orf72^−/−^* mutant retinas

**Figure 8.**
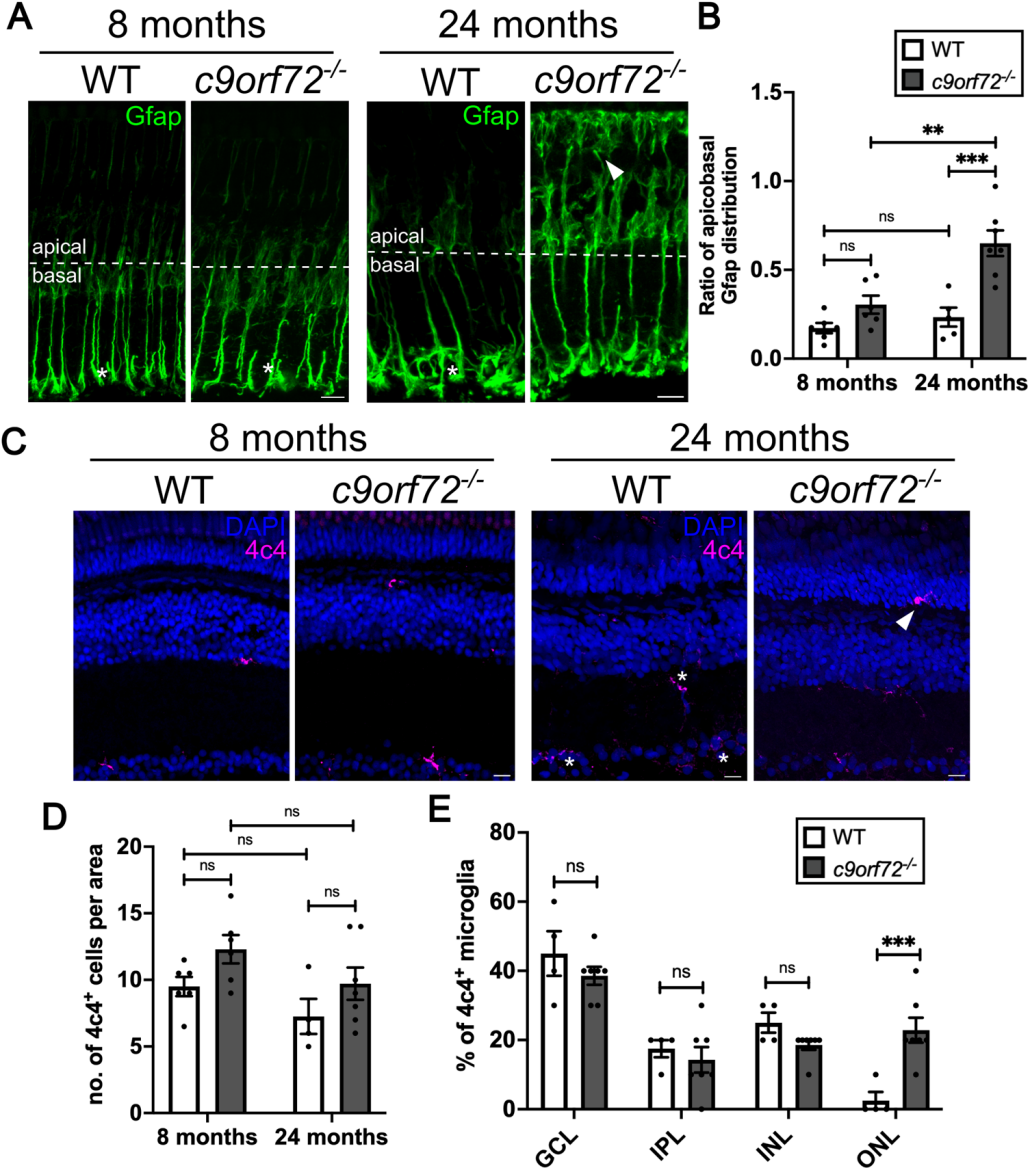
Gliosis phenotypes in *c9orf72^−/−^* deficient retinas. ***A***, Immunostaining for gliosis marker, Gfap (green) in WTS and *c9orf72*-deficient retinal cryosections at 8 and 24 mpf. Nuclei labeled with DAPI (blue). ***B***, Quantification of the mean ratio of apicobasal Gfap distribution across the retina; two-way ANOVA, Šidâk’s multiple-comparisons test; 8 mpf WTS versus 8 mpf *c9orf72^−/−^ p* = 0.5129; 8 mpf WTS versus 24 mpf WTS *p* = 0.9772; 8 mpf *c9orf72^−/−^* versus 24 mpf *c9orf72^−/−^ p* = 0.0013, 24 mpf WTS versus 24 mpf *c9orf72^−/−^ p* = 0.0003. See also Extended Data Figure 8-1. ***C***, Antibody labeling of retinal microglia with 4c4 (magenta), cell bodies labeled with DAPI (blue). ***D***, Quantification of the average number of 4c4^+^ microglia per image; two-way ANOVA, Šidâk’s multiple-comparisons test; 8 mpf WTS versus 8 mpf *c9orf72^−/−^ p* = 0.3989, 8 mpf WTS versus 24 mpf WTS *p* = 0.7408, 8 mpf *c9orf72^−/−^* versus 24 mpf *c9orf72^−/−^ p* = 0.4462, 24 mpf WTS versus 24 mpf *c9orf72^−/−^ p* = 0.6282. See also Extended Data Figure 8-2. ***E***, Quantification of the proportion of 4c4^+^ microglia observed in the ganglion cell layer (GCL), inner plexiform layer (IPL), inner nuclear layer (INL), and the outer nuclear layer (ONL) at 24 mpf; two-way ANOVA, Šidâk’s multiple-comparisons test; GCL, *p* = 0.5932; IPL, *p* = 0.9466; INL, *p* = 0.5932; ONL, *p* = 0.0008. *n* = 5, WTS retinas; *n* = 6, *c9orf72^−/−^* retinas. Scale bar, 10 µm.

As we observed neuronal loss in the retina, we asked whether there were also signs of glial activation (Bringmann et al., 2006; Thomas et al., 2016), similar to what is observed in the aging retina (Martins et al., 2022). To investigate whether *c9orf72* deficiency impacts retinal glia, we performed immunostaining for markers of Müller glia (MG), the principal macroglia of the retina, as well as microglia. In the adult retina, a characteristic sign of MG gliosis is the upregulation and apical redistribution of Gfap (Fernández-Sánchez et al., 2015; Thomas et al., 2016; Graca et al., 2018). Hence, we compared the apicobasal localization of Gfap staining in WTS and *c9orf72*-deficient retinas at 8 and 24 months (Fig. 8*A*,*B*; Extended Data Fig. 8-1). In WTS retinas at both timepoints, Gfap immunoreactivity was confined predominantly to the basal end-feet of the MG (Fig. 8*A*, asterisks). In contrast, in *c9orf72^−/−^* mutants, we observed Gfap staining extending toward the photoreceptors in the outer retina (Fig. 8*A*, arrowhead). Quantification of apicobasal distribution of Gfap at 8 mpf showed no significant difference between WTS and *c9orf72^/−^* mutants (Fig. 8*A*,*B*; *p* = 0.5129; Šídák’s ANOVA; *n* = 6). However, at 24 mpf, the mean apicobasal ratio of Gfap staining in *c9orf72^−/−^* mutants was 2.8-fold higher than WTS (Fig. 8*A*,*B*; *p* = 0.003; Šídák’s ANOVA; *n* = 5–6). This illustrates the apical upregulation and redistribution of Gfap, indicative of reactive gliosis, in the aged *c9orf72^−/−^* retina.

10.1523/JNEUROSCI.2128-23.2024.f8-1 Figure 8-1 **Gfap quantification.** Example of 3D segmentation of Gfap antibody staining in 24-month post fertilisation (mpf) retinal cryosections used for analysis of Gfap distribution along the apicobasal axis. IMARIS was used to quantify the volume of Gfap-staining in the apical (yellow) vs basal (magenta) half of the retina to obtain the ratio of apical:basal Gfap abundance. Left panel: WT; right panel: *c9orf72*. Download Figure 8-1, TIF file.

10.1523/JNEUROSCI.2128-23.2024.f8-2 Figure 8-2 Microglial localisation is unchanged in *c9orf72^-/-^* deficient retinas at 8mpf. A) Quantification of the proportion of 4c4^+^ microglia observed in the ganglion cell layer (GCL), inner plexiform layer (IPL), inner nuclear layer (INL), outer plexiform layere (OPL) and the outer nuclear layer (ONL) at 8mpf; Two-way ANOVA, Šidâk's multiple comparisons test; GCL, p = 0.3830; IPL, p = 0.6354; INL: p = 0.2038; OPL: p = 0.9818 and ONL: p = 9989; n = 6 fish per genotype. B) Example of amoeboid and ramified 4c4^+^ microglial morphologies in the 24mpf-old retinas. Scale bars, 10  µm. C) Quantification of the number of amoeboid vs ramified 4c4^+^ microglia at 24mpf in WTS vs *c9orf72^-/-^*. Amoeboid: p = 0.3762 and ramified: p = 0.9979, two-tailed unpaired t-test, n = 4-6 fish per genotype. Download Figure 8-2, TIF file.

Next, to determine whether C9orf72-deficient retinas exhibited further signs of neuroinflammation, we investigated the localization, morphology, and numbers of microglia using the established zebrafish microglia marker, 4c4. Absolute numbers of 4c4-positive microglia were unchanged between genotypes at either timepoint. However, the proportion of microglia located in the outer retina versus the basal regions at 24 hpf was ninefold higher (Fig. 8*E*; *p* = 0.0008; Šídák’s ANOVA; *n* = 5–6) in *c9orf72^−/−^* mutants compared with WTS controls, consistent with the global photoreceptor degeneration phenotypes (Fig. 8*E*). This was not evident at 8 mpf (Extended Data Fig. 8-2*A*). However, we did not detect 4c4-positive cells in close proximity to the displaced Rho-positive photoreceptors (data not shown) or obvious morphological signs of microglial activation (i.e., ramified vs amoeboid) in *c9orf72^−/−^* mutant retinas (Extended Data Fig. 8-2*B*,*C*). Overall, we observed signs of glial activation in MG and microglia in the absence of C9orf72 function, consistent with a general degeneration phenotype in the retina, which becomes more apparent with age.

## Discussion

*C9ORF72* hexanucleotide repeat expansions are the most common cause of both familiar and sporadic ALS/FTD. Nevertheless, it is unclear if *C9ORF72* LoF, DPR/RNA foci GoF, or a synergy of both is causative for disease. Here, we have established a stable *c9orf72*-deficient zebrafish line to understand the impact of *C9ORF72* LoF in an adult vertebrate in vivo system. Our LoF *c9orf72^−/^*^−^ mutants had conventional development and lived for 24months without any overt signs of ill health. Progressive neurodegeneration was restricted to the retina and comparatively absent from the spinal cord, suggesting that *C9ORF72* deficiency alone is not sufficient to induce a neurodegenerative phenotype in the spinal cord, as demonstrated from mammalian studies (O’Rourke et al., 2016; Dong et al., 2021).

### Comparison with other *c9ORF72*-deficient models

Contrary to our findings, previous reports in the field described *c9orf72*-deficient zebrafish models with comparatively severe phenotypes of axonopathy and early larval lethality (Ciura et al., 2013; Butti et al., 2021). However, both of these models were based on RNA interference which can have potential toxic off-target effects (Robu et al., 2007; Kok et al., 2015). These toxic side effects classically present as neuronal apoptosis, and thus neurodegenerative studies in zebrafish utilizing RNA interference are difficult to interpret, unless validated with a traditional loss of function stable mutant (Kok et al., 2015; Stainier et al., 2017). Furthermore, the discrepancy between stable mutant and RNAi phenotypes is unlikely due to our mutant allele being hypomorphic. Our stable mutation is present within an early exon of *c9orf72*’s single transcript, ablates a key domain, and activates nonsense mediated decay of the mutant mRNA. As zebrafish *c9orf72* lacks any direct paralogs, its inhibition should not activate the transcriptional adaptation pathways which has previously explained phenotypic discrepancies between the RNA interference-based models and their respective stable loss of function mutants (Rossi et al., 2015; El-Brolosy et al., 2019). Additional traditional CRISPR/Cas9 loss of function *c9orf72* zebrafish mutants have also been reported without any accounts of impacted viability (Hruscha et al., 2013; Rounding, 2018). Unexpectedly, our aged mutants did not exhibit similar microglial activation and an inflammatory profile in the wider CNS as described in mouse models (Burberry et al., 2016; O’Rourke et al., 2016; Balendra and Isaacs, 2018). This may be due to a fundamental difference in microglial function between mammals and fish (Geirsdottir et al., 2019).

### Retinal pathology and *c9orf72*

Given the lack of neuroinflammation within the wider CNS, the gliosis, neuronal loss, and degeneration within the retinal tissue of our aged zebrafish *c9orf72^−/−^* was unexpected. Studies in other vertebrate models of C9ORF72 deficiency did not characterize the retina, as such we do not know if they also develop retinal phenotypes. It will be highly prudent to determine how common retinal degeneration is within other C9ORF72-deficient vertebrate models, such as the mouse or rat (Balendra and Isaacs, 2018; Dong et al., 2021) to determine whether retinal pathology is a universal feature of *C9ORF72* loss of function. The retinal phenotypes show an unexpected role for *C9ORF72* in homeostasis of the retina and demonstrate that C9ORF72 deficiency alone can induce spontaneous neurodegeneration, which is relevant for *C9ORF72* linked ALS/FTD. Of note, TDP43 pathology, as well as C9ORF72-specific DPR pathology, has been described within the retina of *C9ORF72* linked ALS/FTD patients (Fawzi et al., 2014; Dijkstra et al., 2021). In the latter, each pathology is found in distinct areas of the retina (OPL and INL, respectively), in a similar fashion to the wider CNS where TDP43 and DPR aggregates are not found in the same neurons (Mori et al., 2013; Dijkstra et al., 2021). Several other ALS linked genes are also associated with neurodegenerative eye diseases (Rezaie et al., 2002; Kawase et al., 2012; Minegishi et al., 2013; Bailey et al., 2016; Nguyen et al., 2018). *TBK1*, *OPTN*, and *ATXN2* variants all cause familial ALS but also glaucoma (Rezaie et al., 2002; Kawase et al., 2012; Minegishi et al., 2013; Bailey et al., 2016; Nguyen et al., 2018). Furthermore, variants in *NEK1* and *C21ORF2* are causative for both ALS and retinitis pigmentosa (Nguyen et al., 2018; Hikoya et al., 2022; Shinbashi et al., 2023). However, C9ORF72’s precise function in retinal neurons is currently not clear.

Interestingly, our analysis of neuronal numbers in *c9orf72 ^−/−^* mutants revealed a variable onset of degeneration between different neuronal subtypes. A decrease in interneurons such as ACs and BPCs was already apparent from 8 mpf and significantly reduced over time. In contrast, other neurons such as RGCs and photoreceptor subtypes began to noticeably decline in number at the later timepoint of 24 mpf. It remains to be determined whether this results from differential sensitivity to C9orf72 deficiency within particular cell types or if C9orf72 acts in a cell type-specific manner, leading to secondary degeneration of other cells. Given the limited assessment of the retina in other models of C9ORF72 deficiency, and our limited knowledge of levels of C9orf72 expression in specific retinal cell types, it is difficult to know exactly the source of the observed degeneration (Balendra and Isaacs, 2018).

In addition to decreased rod photoreceptor numbers with age, we identified basally translocated Rho staining in rod photoreceptors in the *c9orf72^−/−^* retina (Yin et al., 2012). C9ORF72 has been implicated in intracellular trafficking by, for example, interacting with Rab proteins which can influence motor proteins involved in intracellular transport of proteins (Hammer and Wu, 2002). The mislocalization of rhodopsin from outer segments to the cell body and neurites of rods may point to a potential involvement of C9orf72 in opsin trafficking. Indeed, similar rhodopsin mistrafficking phenotypes were reported in mice lacking unconventional Myosin 1C (MYO1C), a motor protein, as well as in zebrafish deficient in Centrosomal Protein 290 (Cep290), a cilliary protein found in the connecting cillium between photoreceptor inner and outer segments (Lessieur et al., 2019; Solanki et al., 2021). However, we did not observe any evidence of opsin mislocalization in the mutants at 8 mpf, and the decrease in rod numbers was only evident in the aged 24 mpf retinas; it is unlikely that opsin trafficking is defective due to the loss of C9orf72 directly. Instead, it is highly plausible that the global degeneration of the surrounding cells creates a toxic environment, thereby subsequently inducing degeneration in the photoreceptors. This would lead to photoreceptor outer segment damage and eventual opsin mislocalization, which is commonly seen in other models of retinal degeneration (Hammer and Wu, 2002; Lessieur et al., 2019; Solanki et al., 2021). It will be important to determine whether retinal changes, such as thinning, can be used as a reliable early indicator of neurodegeneration in the wider CNS, as well as furthering our understanding of C9ORF72 physiological and pathological functions.

Retinal thinning is a key feature of our *c9orf72* mutant zebrafish, which is also present in ALS patients that do not otherwise present with ophthalmic disease (Ringelstein et al., 2014; Mukherjee et al., 2017; Cerveró et al., 2021). Ocular assessment is currently absent from the diagnostic pipeline in clinics, and hence confirming the presence of similar retinal phenotypes in mammalian model organisms may provide critical evidence for the need for thorough ocular tests as part of ALS diagnosis. As the retina is part of the CNS, most neurodegenerative diseases that affect neurons in the brain or spinal cord also impact retinal neurons. For example, retinal thinning and neuronal loss are common features presented by Alzheimer’s disease patients (Coppola et al., 2015) even in early stages of the disease and despite its progressive nature. The same is also true for Huntington’s disease (Andrade et al., 2016), further highlighting the potential usefulness of retinal assessment for the diagnosis of other neurodegenerative diseases (den Haan et al., 2017; Chrysou et al., 2019; Guidoboni et al., 2020).

### Conclusions

Our study shows novel pathology in the context of *c9orf72* deficiency in vivo, namely, retinal neurodegeneration, demonstrating *c9orf72* LoF can trigger neuronal loss spontaneously. Additionally, we have characterized a stable *c9orf72*^−/−^ zebrafish, which is highly valuable to understand not only the mechanism of retinal degeneration we have described, but also to study synergistic effects of ALS risk factors, mutations, and pathways potentially causative of neurodegenerative disease in a rapid and cost-effective in vivo vertebrate model system.
