# Supplementary figures and images for: C9ORF72 Deficiency Results in Neurodegeneration in the Zebrafish Retina
Source: J Neurosci. 2024 Apr 24;44(25):e2128232024. doi: 10.1523/JNEUROSCI.2128-23.2024 (PMC11209673; doi:10.1523/JNEUROSCI.2128-23.2024)

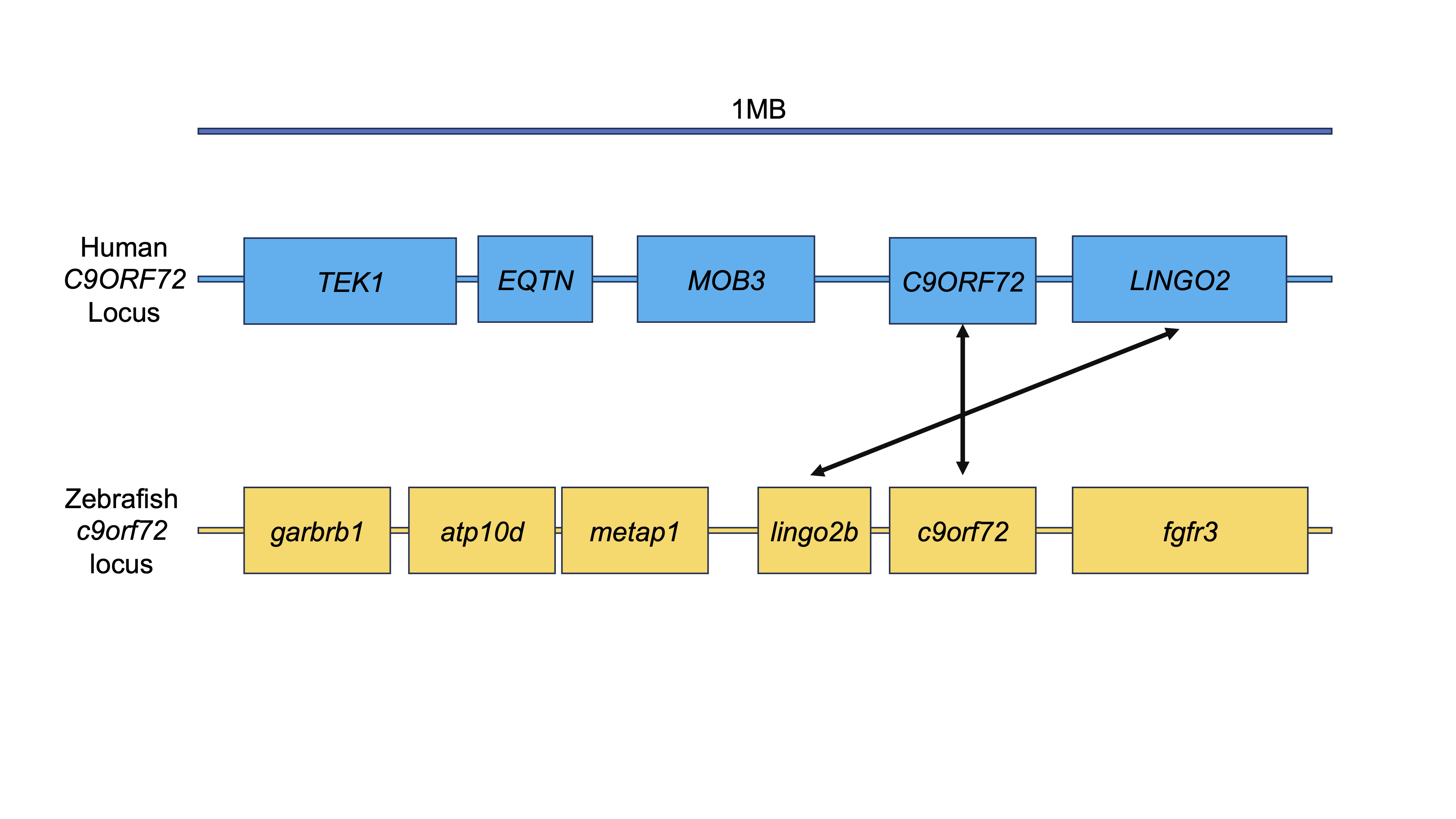

Supplement: Figure 1-1 — Schematic of conserved gene synteny between the human C9ORF72 and zebrafish c9orf72. In both species the genes encoding C9ORF72 and LINGO2 are located within 0.5mb of each other on the same chromosome. Download Figure 1-1, TIF file. [file jneuro-44-e2128232024-s001.tif]

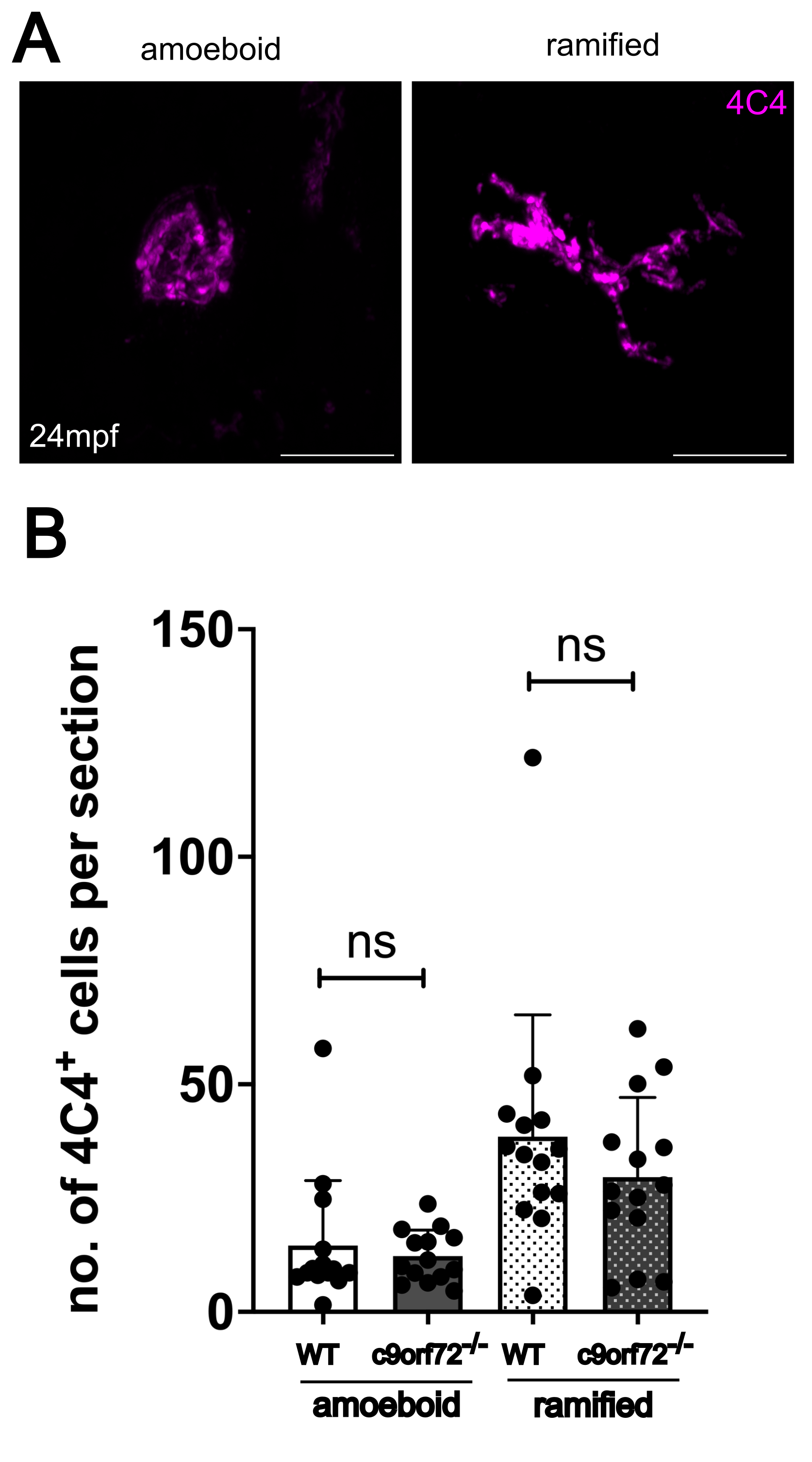

Supplement: Figure 3-1 — Morphology of spinal cord microglia is not altered in c9orf72-/-. A) Example images of amoeboid and ramified spinal cord microglia at 24mpf. Scale bar: 10 mm. B) Quantification of Figure 3A, taking into account the morphological differences of amoeboid (WT: 14.57, c9orf72-/-: 12.24) and ramified (WT: 38.49, c9orf72-/-: 29.62) microglia, which is unaltered in c9orf72-/- mutants. Unpaired, two-tailed and parametric t-test, amoeboid: p = 0.5765, n-WT:13, n- c9orf72-/-:14, ramified: p = 0.3087, n-WT:13, n- c9orf72-/-:14. Download Figure 3-1, TIF file. [file jneuro-44-e2128232024-s002.tif]

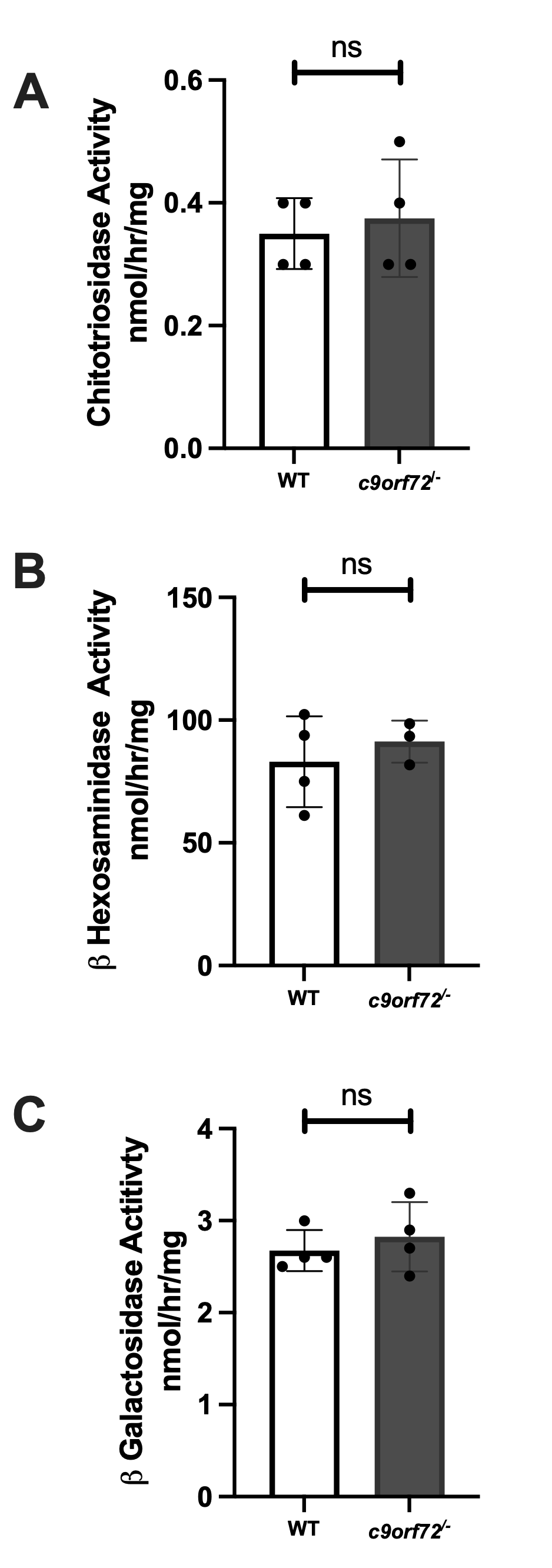

Supplement: Figure 3-2 — Lysosomal enzyme activities are not significantly altered between c9orf72-/- brains and WT controls. Measurements of different enzyme activities from whole brain homogenates revealed no statistical difference in activity between genotypes. These included Chitotriosidase activity (2A, p = 0.6704) a general marker of neuroinflammation and potential ALS biomarker. The lysosomal enzyme enriched in microglia, b Hexosaminidase (2B, p = 0.5156) and the lysosomal enzyme b Galactosidase (2C, p = 0.5188). Unpaired, two-tailed and parametric t-test, n = 4 for all. These data indicate there is minimal neuroinflammation occurring in the brain. Download Figure 3-2, TIF file. [file jneuro-44-e2128232024-s003.tif]

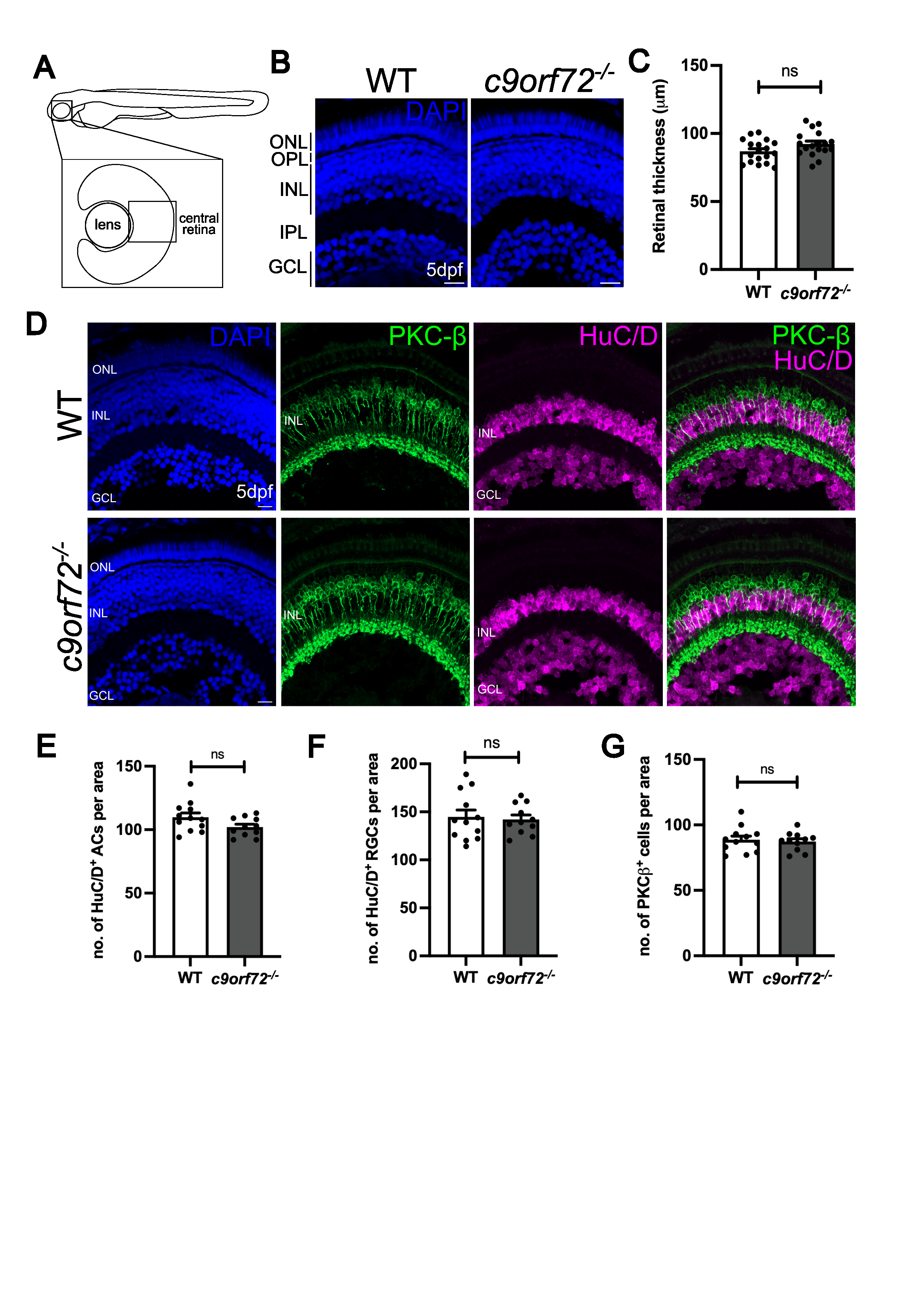

Supplement: Figure 4-1 — Inner retinal development is normal in c9orf72-/- mutants. A) Schematic diagram of 5-day post fertilisation (dpf) zebrafish retina, highlighting the central retinal region used for analysis. B) DAPI (blue) staining showing the nuclear layers of the WT and c9orf72-/- larval retina and overall central retinal thickness. C) Quantification of mean thickness of WT and c9orf72-/- retinas; unpaired t-test; p = 0.0704. D) Antibody staining for bipolar cell marker, (PKC-b, green), amacrine and retinal ganglion cells (HuC/D, magenta) and nuclei stained with DAPI (blue) in WT and c9orf72-/- retinas. E) Quantification of number of HuC/D+ amacrine cells in INL per 100 mm x 100 × 10 mm ROI; unpaired t-test; p = 0.0726. F) Quantification of number of HuC/D+ retinal ganglion cells in GCL per 100 mm x 100 x10 mm ROI; unpaired t-test; p = 0.7723. G) Quantification of number of PKC-b+ bipolar cells in INL per 100 mm x 100 x10 mm ROI; unpaired t-test; p = 0.7024; n = 11-12 larvae per genotype; Scale bars, 10 mm. Download Figure 4-1, TIF file. [file jneuro-44-e2128232024-s004.tif]

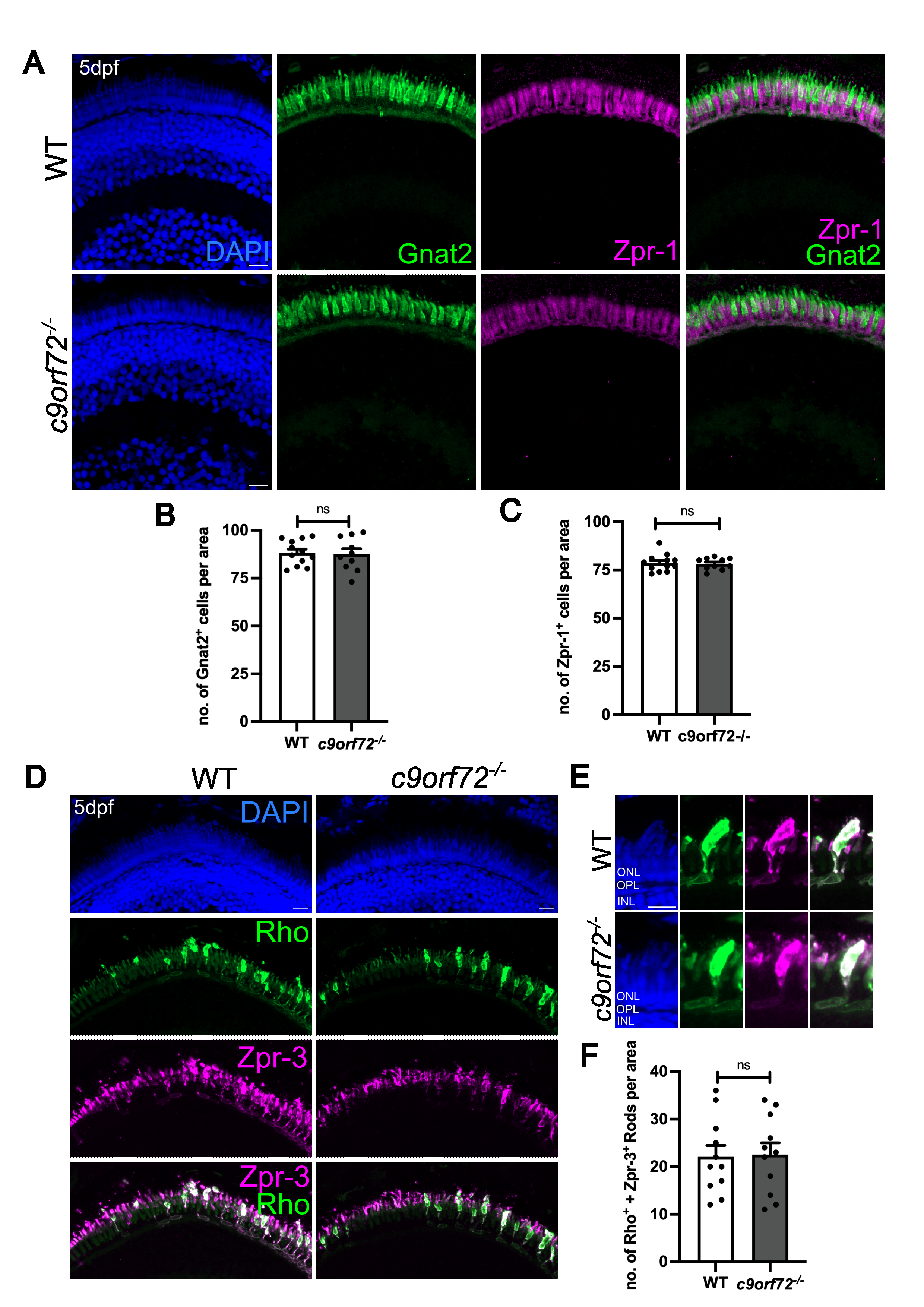

Supplement: Figure 4-2 — Rod and cone photoreceptors do not exhibit developmental defects in c9orf72-/- mutants. A) Immunostaining for pan-cone marker, Gnat2 (green) and double cone marker, Zpr-1 (magenta) in WT and c9orf72-deficient retinal cryosections at 5dpf. Nuclei labelled with DAPI (blue). B) Quantification of mean number of Gnat2-positive cone photoreceptors in WT and c9orf72-deficient retinas;100 mm x 100 × 10 mm ROI; unpaired t-test, p = 0.8238. C) Quantification of mean number of Zpr-1-positive cone photoreceptors in WT and c9orf72-deficient retinas;100 mm x 100 × 10 mm ROI; unpaired t-test, p = 0.8209. D) Antibody staining for Rhodopsin (Rho; green) and rod/double cone marker Zpr-3 (magenta). E) Close up of individual Rho + Zpr-3-expressing rods in WT and mutants. F) Quantification of mean number of Rho + Zpr-3+ rod photoreceptors in WT and c9orf72-deficient retinas;100 mm x 100 mm ROI; unpaired t-test, p < 0.0001. n = 10-12 retinas per genotype. Scale bars, 10 mm. Download Figure 4-2, TIF file. [file jneuro-44-e2128232024-s005.tif]

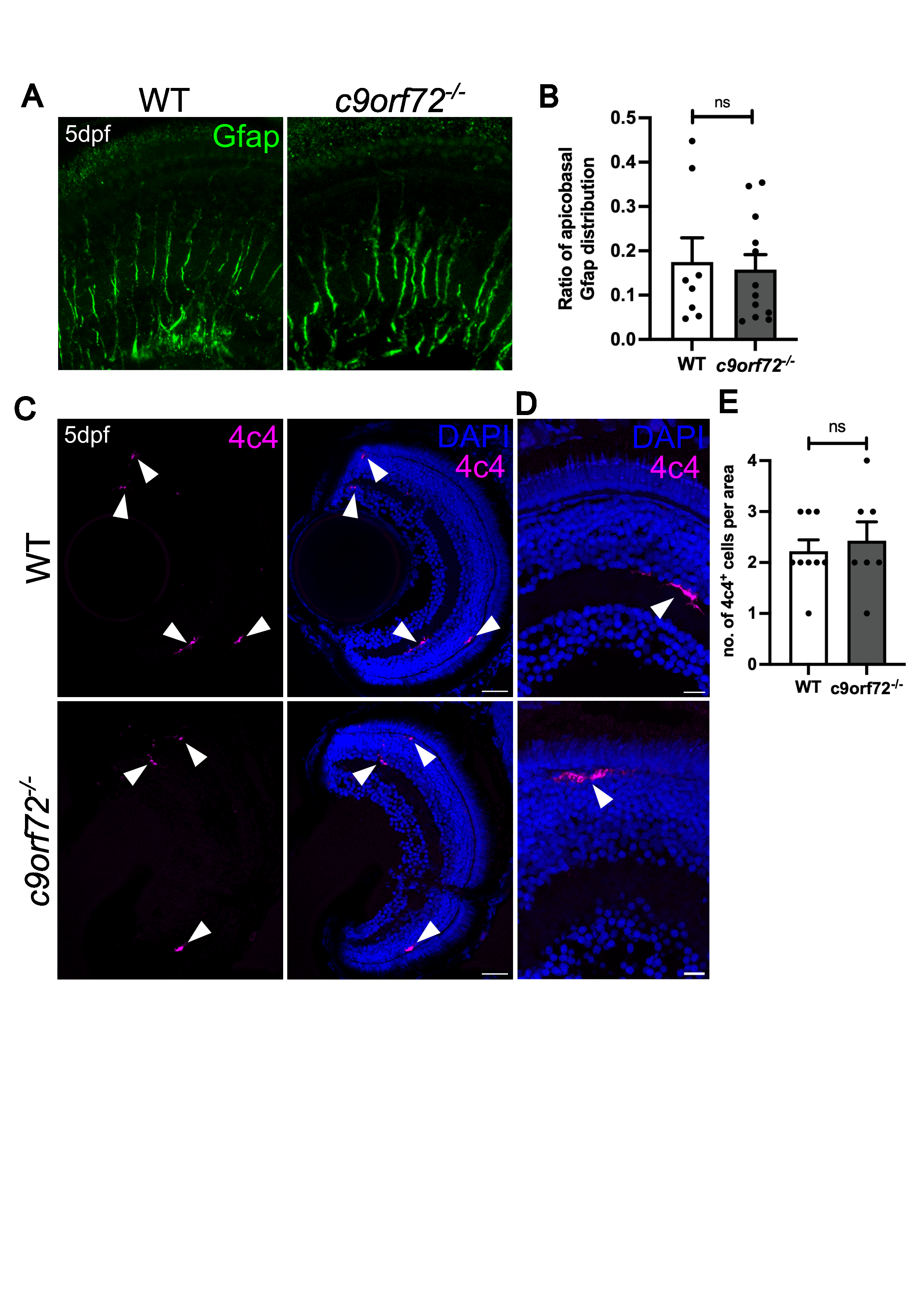

Supplement: Figure 4-3 — Glial development is unaffected in c9orf72-/- deficient retinas. A) Immunostaining for gliosis marker, Gfap (magenta) in WT and c9orf72-deficient retinal cryosections at 5dpf. Nuclei labelled with DAPI (blue). B) Ratio of mean Gfap distribution in apical versus basal regions of the retina; unpaired t-test, p = 0.7791. C) Antibody labelling of retinal microglia with 4c4 (magenta), cell bodies labelled with DAPI (blue). D) Higher magnification image of 4c4 and DAPI staining. E) Quantification of the average number of 4c4+ microglia per image; unpaired t-test; p = 0.6226; Scale bars, 10 mm. Download Figure 4-3, TIF file. [file jneuro-44-e2128232024-s006.tif]

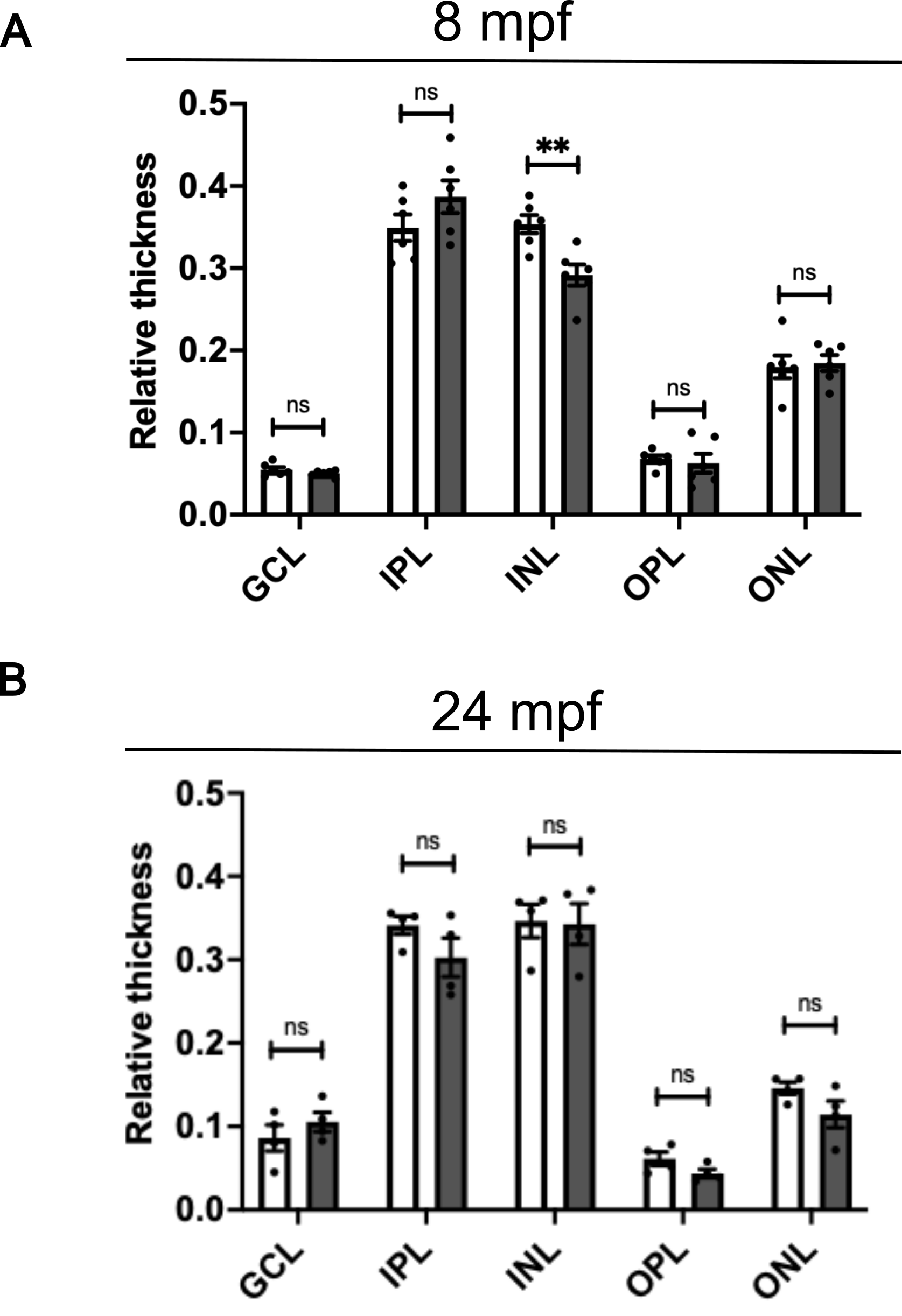

Supplement: Figure 4-4 — Retinal layer become proportionally thinner in c9orf72-/- mutants with age. A) Quantification of the thickness ratios of retinal layers compared to the overall retinal thickness in WTS and c9orf72-/- mutants at 8months post fertilisation (mpf). Ganglion cell layer (GCL): p = 0.9995, inner plexiform layer (IPL): p = 0.1333, inner nuclear layer (INL): p = 0.0025, outer plexiform layer (OPL): p = 0.9990 and outer nuclear layer (ONL): p = 0.9994. Two-way ANOVA, Šídák's multiple comparisons test; n = 6 fish per genotype. B) Quantification of the thickness of each retinal layers compared to the overall retinal thickness in WTS and c9orf72-/- mutants at 24mpf. GCL: p = 0.9172, IPL: p = 0,3725 INL:p > 0.9999, OPL: p = 0.9398 and ONL:p = 0.5942. Two-way ANOVA, Šídák's multiple comparisons test; n = 5-6 fish per genotype. Download Figure 4-4, TIF file. [file jneuro-44-e2128232024-s007.tif]

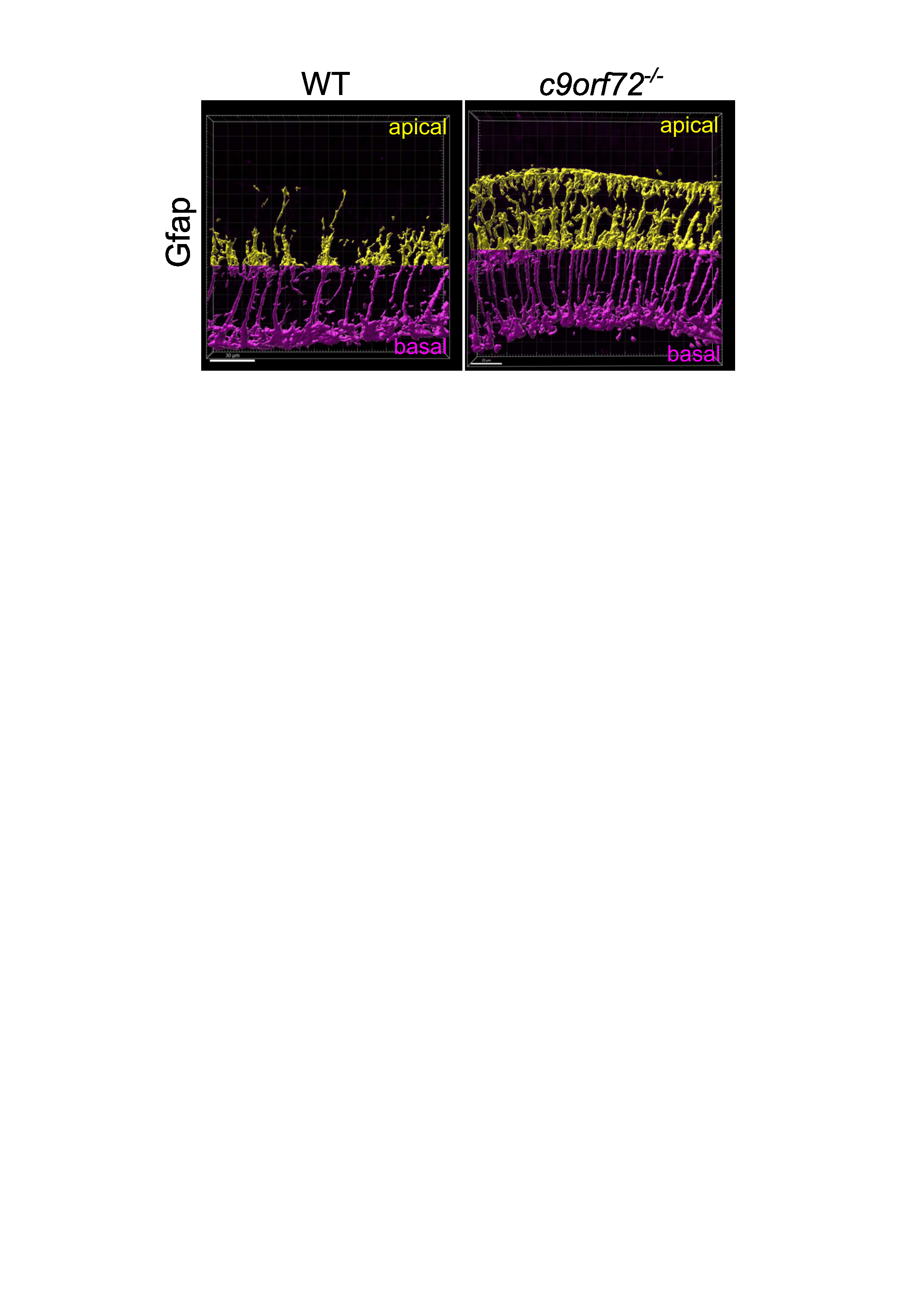

Supplement: Figure 8-1 — Gfap quantification. Example of 3D segmentation of Gfap antibody staining in 24-month post fertilisation (mpf) retinal cryosections used for analysis of Gfap distribution along the apicobasal axis. IMARIS was used to quantify the volume of Gfap-staining in the apical (yellow) vs basal (magenta) half of the retina to obtain the ratio of apical:basal Gfap abundance. Left panel: WT; right panel: c9orf72. Download Figure 8-1, TIF file. [file jneuro-44-e2128232024-s008.tif]

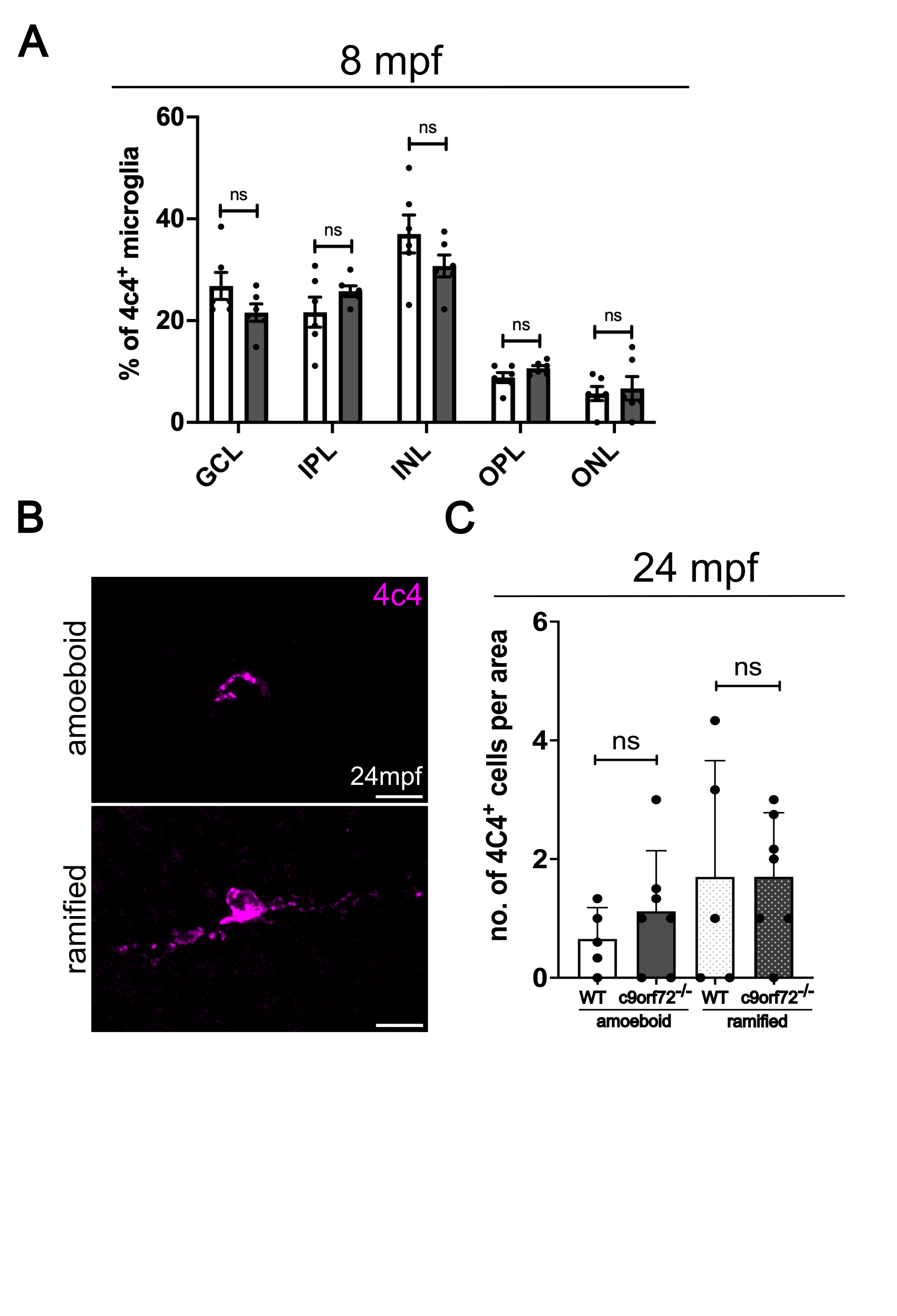

Supplement: Figure 8-2 — Microglial localisation is unchanged in c9orf72-/- deficient retinas at 8mpf. A) Quantification of the proportion of 4c4+ microglia observed in the ganglion cell layer (GCL), inner plexiform layer (IPL), inner nuclear layer (INL), outer plexiform layere (OPL) and the outer nuclear layer (ONL) at 8mpf; Two-way ANOVA, Šidâk's multiple comparisons test; GCL, p = 0.3830; IPL, p = 0.6354; INL: p = 0.2038; OPL: p = 0.9818 and ONL: p = 9989; n = 6 fish per genotype. B) Example of amoeboid and ramified 4c4+ microglial morphologies in the 24mpf-old retinas. Scale bars, 10 µm. C) Quantification of the number of amoeboid vs ramified 4c4+ microglia at 24mpf in WTS vs c9orf72-/-. Amoeboid: p = 0.3762 and ramified: p = 0.9979, two-tailed unpaired t-test, n = 4-6 fish per genotype. Download Figure 8-2, TIF file. [file jneuro-44-e2128232024-s009.tif]
